# Comparability of Steroid Collision Cross Sections Using Three Different IM-HRMS Technologies: An Interplatform Study

**DOI:** 10.1021/jasms.2c00196

**Published:** 2022-09-01

**Authors:** Max L. Feuerstein, Maykel Hernández-Mesa, Andrea Kiehne, Bruno Le Bizec, Stephan Hann, Gaud Dervilly, Tim Causon

**Affiliations:** † Department of Chemistry, Institute of Analytical Chemistry, University of Natural Resources and Life Sciences, Vienna, Muthgasse 18, 1190 Vienna, Austria; ‡ Oniris, INRAE, LABERCA, 44300 Nantes, France; § Bruker Daltonics GmbH & Co. KG, 28359 Bremen, Germany

## Abstract

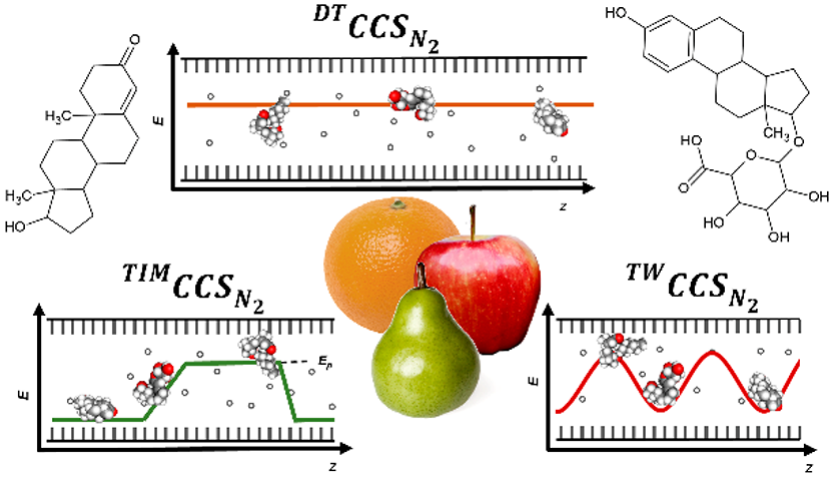

Steroids play key roles in various biological processes and are characterized by many isomeric variants, which makes their unambiguous identification challenging. Ion mobility-mass spectrometry (IM-MS) has been proposed as a suitable platform for this application, particularly using collision cross section (*CCS*) databases obtained from different commercial IM-MS instruments. *CCS* is seen as an ideal additional identification parameter for steroids as long-term repeatability and interlaboratory reproducibility of this measurand are excellent and matrix effects are negligible. While excellent results were demonstrated for individual IM-MS technologies, a systematic comparison of *CCS* derived from all major commercial IM-MS technologies has not been performed. To address this gap, a comprehensive interlaboratory comparison of 142 *CCS* values derived from drift tube (DTIM-MS), traveling wave (TWIM-MS), and trapped ion mobility (TIM-MS) platforms using a set of 87 steroids was undertaken. Besides delivering three instrument-specific *CCS* databases, systematic comparisons revealed excellent interlaboratory performance for 95% of the ions with *CCS* biases within ±1% for TIM-MS and within ±2% for TWIM-MS with respect to DTIM-MS values. However, a small fraction of ions (<1.5%) showed larger biases of up to 7% indicating that differences in the ion conformation sampled on different instrument types need to be further investigated. Systematic differences between *CCS* derived from different IM-MS analyzers and implications on the applicability for nontargeted analysis are critically discussed. To the best of our knowledge, this is the most comprehensive interlaboratory study comparing *CCS* from three different IM-MS technologies for analysis of steroids and small molecules in general.

Steroids are a class of cholesterol derivatives and are characterized by a large diversity of isomers that are of interest as even small structural changes can have a huge impact on their biological activity.^1−3^ Due to their role in regulating metabolism, growth, reproductive function, and immune response, steroids are analyzed across a wide range of research fields.^1,2^ Furthermore, the use of exogenous anabolic androgenic steroids (AASs) to enhance growth in livestock production was banned by the European Union (EU),^4^ and various natural and synthetic steroids are prohibited doping agents in competitive sports.^5−7^ Within this context, steroids are analyzed in diverse biological matrices including serum, brain tissue, or urine.^1,6,8^ Relatively lipophilic steroids are metabolized via a two-phase reaction, and typically phase II metabolites (i.e., sulfates and glucuronides) are excreted due to their better solubility.^3^ Steroid phase II metabolites are of major interest when analyzing urine (e.g., for drug testing, in chemical food safety or doping control) due to a high degree of metabolization and secretion.^5,6^ The combination of complex matrices, low concentrations, and large number of possible steroid isomers^8^ demands high-performance methods in terms of selectivity and sensitivity.^6^ Therefore, mass spectrometry (MS) coupled to front-end chromatographic separation such as liquid chromatography (LC) has become one of the key technologies for the analysis of both steroids and their phase II metabolites.^2^

More recently, ion mobility-mass spectrometry (IM-MS) has also gained significant attention for potential application to separate and identify isomeric and isobaric steroids.^5,9,10^ As a standalone technology, IM-MS has been applied for rapid steroid analysis, but its application is limited for the most demanding applications due to the high complexity of biological matrices and by the limited resolution of current IM technology.^1,11^ However, in combination with LC (i.e., LC-IM-MS), the benefits of both analytical platforms can be exploited.^5^ For example, signal-to-noise ratios were improved when IM-filtering was used for mass spectra cleanup.^6^ In addition to increased peak capacity and cleaner fragment spectra,^12^ IM-derived collision cross sections (*CCS*s) have been extensively discussed as a potential ion species-specific descriptor for identity confirmation,^6,13,14^ and some notable studies focused on the potential analysis of steroids using (LC−)IM-MS have been published recently.^5,6,9,10,14−16^ Application of *CCS* as additional identification parameter is of special interest for small molecules that exhibit limited fragmentation or formation of only unspecific fragments.^17,18^ This is indeed the case for many steroids, which leads to ambiguous assignment of isomer identities.^5,6^ Since transitioning into the commercial arena in 2006, several different IM-MS instrument types are now commercially available including high-field asymmetric IM (FAIM-MS), drift tube (DTIM-MS), traveling wave (TWIM-MS), and trapped IM (TIM-MS) technologies.^19−21^ While FAIMS is typically used as a selective IM filter (e.g., in drug screening^22^), time-dispersive techniques such as DTIM-MS and TWIM-MS or confinement-and-release technology (i.e., TIM-MS) can be used as generic IM separators for nontargeted full-scan analysis.^13^ When comparing data derived from these instrument types, the different principles of IM devices are important to highlight. In DTIM, ions are accelerated in a low, uniform electric field and separated in the drift tube containing a neutral buffer gas.^13,23^ Ion separation is analogous in TWIM, but dynamic electric fields are applied to drag the ions through the buffer gas.^20,24^ Finally, TIM involves the spatial trapping of ions by opposing forces of a moving buffer gas flow and an electric field gradient. In this case, ions are released by incrementally lowering the applied potential barrier and exit the TIM separator in inverse order compared to DTIM or TWIM.^21,25^ While the principles differ, these three instrument types all offer a fast and partly orthogonal IM-separation that can improve analytical performance of LC-MS methods,^9,26,27^ allow the application of novel acquisition modes for data independent acquisition^12^ and data dependent acquisition,^27^ and can be externally calibrated for determination of *CCS* values for all detected ions.^23,28,29^

With a focus on database-driven identity confirmation of small molecules, several experimental *CCS* data sets and databases for small molecules have been published in recent years. Besides extensive multiclass databases such as the *CCS* Compendium,^30^ several data sets for certain compound classes are publicly available and include metabolites,^31^ lipids^32^ and steroids.^5,9,10,14^ In addition to experimental *CCS* databases, the number of computationally (*in silico*) predicted *CCS* libraries, either based on structural predictions based on density functional theory calculations^33−35^ or machine learning is also increasing.^8,36,37^ However, a comprehensive evaluation of the comparability of *CCS* derived from different classes of IM-MS instruments is still lacking and assessment of possible differences is mandatory before such databases can be applied across different classes of IM-MS instruments.^19,38^ This evaluation is critically important when considering the key question of “*what does a CCS actually represent?*”—a question that continues to be addressed in fundamental research,^39−41^ despite its increasing use across diverse analytical applications. From a fundamental perspective, *CCS* differs from *m*/*z* information as it is a conditional value derived from an ion’s mobility (*K*) and depends on properties of the ion, such as size or charge state, as well as the buffer gas, temperature, and the field strength-to-pressure ratio.^19^ However, it is well-established that ionization, ion transfer, and ion separation can also influence the observed ion structure and hence the ion’s mobility, e.g., via formation of protomeric isomers,^19,42^ other types of open/closed conformers,^43^ or metastable solvent clusters.^44^ In combination, this influences the comparability of *CCS* values and the question to which extent *CCS* reference values can be established and used independently of the instrument type is still under discussion.^45^ Finally, we note that the external calibration strategies for *CCS* determination employed affect the comparability of *CCS* across laboratories.^13,19^ Of the current major instrument technologies, low-field DTIM-MS presents the closest relationship to fundamental ion mobility theory and is used to generate primary data (i.e., stepped-field method) for the establishment of reference ^*DT*^*CCS*_*N2,ref.*_ values for secondary calibration approaches. As a consequence, this direct link to primary ^*DT*^*CCS*_*N2,ref.*_ values is maintained between a well-characterized reference instrument^23^ and other DTIM-MS instruments using the same set of reference values for single-field calibration. Nevertheless, uncertainties on these reference *CCS* values have to be considered as these are propagated into secondary calibration strategies for determination of *CCS* across all three major instrument types.^23,46^ For these reasons, the true merit of applying *CCS* in steroid analysis demands for a comprehensive study of all major commercial instruments including their prescribed external calibration procedures.

To address these open questions, we undertook a large-scale comparison of three major commercial IM-QTOF instrument classes for application to the analysis of steroids. For this purpose, new ^*DT*^*CCS*_*N2*_ and ^*TIM*^*CCS*_*N2*_ reference data sets were established to complement existing single laboratory and interlaboratory reference ^*TW*^*CCS*_*N2*_ values.^9,14^ To the best of our knowledge, this is the only comprehensive study comparing experimental CCS values for steroids using all three commercially available technologies relevant to this application (i.e., DTIM-MS, TWIM-MS, and TIM-MS).

## Materials and Methods

### Chemicals and Reagents

Stock solutions of steroid standards (100 μg/mL or 1 mg/mL) from Steraloids (Newport, RI, USA), Sigma-Aldrich (St. Louis, MO, USA), and National Measurement Institute (NMI, Pymble, Australia) were stored in ethanol at −20 °C, and several mixed solutions (10 μg/mL) were prepared for LC-IM-MS measurements (see the Supporting Information). For LC-DTIM-MS analysis, LC-MS-grade water from a Milli-Q IQ 7000 purification system equipped with an LC-Pak polisher cartridge (Merck Chemicals and Life Science GmbH, Vienna), and LC-MS-grade acetonitrile (CAN) and formic acid (FA) from Sigma-Aldrich were used for dilutions of standards and preparation of mobile phase. ESI Tune Mix (ESI-L, G1969-85000, Agilent Technologies) along with 0.1 mmol/L HP-0321 from Agilent Biopolymer Reference Kit was used to tune and calibrate the DTIM-MS instrument according to the manufacturer instructions. For LC-TIM-MS measurements, ultrapure water from an ELGA LabWater–water purification system was used along with MS-grade acetonitrile (Biosolve, Netherlands) and MS-grade formic acid (Honeywell Fluka).

### Standards and Sample Preparation

A set of 87 steroids based on a previous interlaboratory comparison of different TWIM-MS systems was adapted for this study.^14^ Pure standard mixtures were prepared with concentrations of 1 or 5 μg/mL for direct infusion and 1 μg/mL for LC-DTIM-MS analysis. Standard mixtures of water-soluble steroids were prepared in 95:5 of 0.1% (v/v) formic acid/acetonitrile, while hydrophobic steroids (e.g., sterol esters) were prepared in 50:50 of 0.1% (v/v) formic acid/acetonitrile. To obtain a well-defined solvent composition, spiked samples and standard mixtures were dried under nitrogen and redissolved in an appropriate final composition ready for analysis. The exact composition of the steroid mixtures is presented in the Supporting Information. Bovine urine samples from adult animals and calves used to study matrix effects were stored in the LABERCA biobank. Urine samples were thawed, diluted 10-fold, and spiked with a mixture containing 69 steroids to a final concentration of 0.5 μg/mL prior to analysis.

### IM-MS Instrumentation, Calibration, and Acquisition

Across the three commercial instrument technologies assessed in this study, the ion transport mechanisms and routine analytical procedures for external *CCS* calibration are known to be inherently different.^13,19^ Recommended acquisition settings and application of routine external *CCS* calibration were used. Details of the underlying theory for DTIM-MS, TWIM-MS and TIM-MS are presented in the Supporting Information. TWIM-MS data sets were reported in two of our previous publications and publicly available data was used for all comparisons.^9,14^ Data from these studies are referred to as the single laboratory^9^ and interlaboratory^18^^*TW*^*CCS*_*N2*_ libraries, respectively. DTIM-MS measurements were performed using an Agilent 6560 IM-QTOFMS instrument equipped with an Agilent Dual Jet Stream ESI source using various acquisition methods and conditions. Prior to measurements, the instrument was tuned and calibrated using manufacturer recommendations (ESI-L, G1969-85000, Agilent Technologies). Both stepped-field and single-field measurements were carried out following the method of Stow et al.^23^ Briefly, ^*DT*^*CCS*_*N2*_ determined using the stepped-field method as well as single-field operation with either standard or 4-bit multiplexing settings. Long-term repeatability of ^*DT*^*CCS*_*N2*_ was evaluated by reanalyzing steroids 9 months after the first measurements using 4m acquisition.

For generation of a ^*TIM*^CCS_*N2*_ data set, an Elute UHPLC was coupled to a timsTOF Pro (Bruker Daltonics, Bremen Germany) using an ESI source and the same LC conditions, and columns were used as described for DTIM-MS analysis except for the LC flow rate (600 μL/min) and injection volume (5 μL) applied. This instrument platform yielded an IM-resolving power of *R*_*p*_ ∼ 60–80 under the conditions applied in this study. The timsTOF Pro was operated using the Bruker OTOFcontrol (6.2) software along with HyStar (5.1) software. Prior to analysis, the instrument was mass calibrated with sodium formate clusters (10 mM in 50:50 2-propanol/water) and ^*TIM*^*CCS*_*N2*_ was calibrated using ions from Agilent ESI-L Tune Mix via a linear function. In addition to external calibration before analysis, automatic postrun recalibration was used. Spiked urine samples (see above) were analyzed using the same settings as used for standard mixtures. Matrix effects of urine on determined ^*DT*^*CCS*_*N2*_ and ^*TIM*^*CCS*_*N2*_ were determined by analyzing 69 steroids spiked into urine samples with final concentrations of 0.5 mg/mL. Full method parameters for all applied methods are found in the Supporting Information.

### Data Processing and Evaluation

For DTIM-MS, Agilent IM-MS Browser 10.0 was used for single-field calibration, evaluation of stepped-field ^*DT*^*CCS*_*N2*_, and manual inspection when required. Agilent MassHunter Mass Profiler 10.0 was used for feature extraction (peak picking) of triplicate LC-DTIM-MS measurements following single-field calibration and direct infusion. PNNL Preprocessor 3.0 (2021.04.21) was used for demultiplexing and data preprocessing steps.^47^

For TIM-MS, Bruker TASQ software (version 2021) was used to analyze all data acquired using the timsTOF Pro. This included automatic recalibration, generation of high-resolution extracted ion chromatograms (EICs), feature detection (peak picking), and ^*TIM*^*CCS*_*N2*_ calculation. Full details of used data processing can be found in the Supporting Information. Exported data was restructured, analyzed, and visualized using Microsoft Office (Excel and Powerpoint) and R (4.1.2)^48^ together with RStudio (2021.9.1.372)^49^ (see the Supporting Information).

## Results and Discussion

For a comprehensive evaluation of reproducibility for *CCS*_*N2*_ determination using different instrument classes and methods, reference ^*DT*^*CCS*_*N2*_ values were generated. For this purpose, stepped-field and secondary single-field methods with either standard operation mode or 4-bit multiplexing were applied. In the used DTIM-QTOFMS instrument, ions are accumulated in a trapping funnel followed by release and DTIM-separation once per measurement cycle (standard operation). The multiplexed operation increases ion utilization efficiency, increases working range and reduces instrument saturation including the minimization of space-charge effects in comparison to standard operation.^50^ In total, 135 single-field ^*DT*^*CCS*_*N2*_ values and 102 stepped-field ^*DT*^*CCS*_*N2*_ values were determined with the DTIM-MS platform and were used as the basis of interplatform *CCS* comparisons (see the Supporting Information). Precision under conditions of repeatability for measurements of ^*DT*^*CCS*_*N2*_ and ^*TIM*^*CCS*_*N2*_ were both excellent (i.e., average RSD < 0.2%), and effects of bovine urine matrix were negligible. In addition, determined ^*DT*^*CCS*_*N2*_ values were in good agreement with recently published data sets by Velosa et al.^11^ and Davis et al.^5^ with average differences <0.5% for a small number of steroids determined (see Table S2). Similar figures of merit for ^*TW*^*CCS*_*N2*_ determination of steroids have been previously reported.^6,14^

### Comparisons of ^*DT*^*CCS*_*N2*_, ^*TW*^*CCS*_*N2*_, and ^*TIM*^*CCS*_*N2*_ Data Sets

In addition to single IM-MS technology interlaboratory studies, some comparisons across two instrument technology classes for small molecules were performed previously. For example, differences between ^*TIM*^*CCS*_*N2*_ and ^*DT*^*CCS*_*N2*_ of 0.53% to 2.1% have been determined for plant metabolite data sets,^29^ and mean percentage errors of ^*TW*^*CCS*_*N2*_ compared to ^*DT*^*CCS*_*N2*_ were 1.0% and 1.1% for [M+H]^+^ and [M+Na]^+^ ions, but deviations of up to 6.2% were reported for some ions in the first study that compared the commercial Agilent DTIM-MS with Waters TWIM-MS for small molecule applications.^38^ Based on the new data sets and the existing ^*TW*^*CCS*_*N2*_ database,^14^ a new comprehensive interplatform and interlaboratory *CCS*_*N2*_ database for steroids and phase II metabolites containing 142 ions was created (see the Supporting Information). The correlation between individual data sets from the three different instruments was investigated, and the appearance of outliers and systematic differences observed for [M–H]^−^, [M+H]^+^, and [M+Na]^+^ species of steroids was further studied. To compare all data sets, Pearson correlation coefficients (*r*) were determined. Coefficients of *r* = 0.9949 and *r* = 0.9953 were obtained when comparing ^*TW*^*CCS*_*N2*_ and ^*TIM*^*CCS*_*N2*_ with ^*DT*^*CCS*_*N2*_ as the reference and *r* = 0.9989 when directly comparing ^*TW*^*CCS*_*N2*_ with ^*TIM*^*CCS*_*N2*_ (Figure S1) indicating the good agreement between *CCS* data sets from all three instrument types. Single-field ^*DT*^*CCS*_*N2*_ data from multiplexed operation was used as reference to calculate bias against results from TIM-MS and TWIM-MS instruments. The histograms of the absolute bias (in %) of ^*TW*^*CCS*_*N2*_ and ^*TIM*^*CCS*_*N2*_ and bias distribution visualized as violin plots for comparisons of ^*TW*^*CCS*_*N2*_ and ^*TIM*^*CCS*_*N2*_ data sets with ^*DT*^*CCS*_*N2*_ (4m) values are shown in Figure S2. These comparisons illustrate at first glance the lower absolute bias observed between ^*TIM*^*CCS*_*N2*_ and ^*DT*^*CCS*_*N2*_ (mean = 0.47% ± 0.70%, 95th percentile = 1.03%) than that between ^*TW*^*CCS*_*N2*_ and ^*DT*^*CCS*_*N2*_ (mean = 0.82% ± 0.76%, 95th percentile = 1.92%). Two further observations can be made from these comparisons: (1) a systematic negative bias irrespective of the ion species was observed between ^*TW*^*CCS*_*N2*_ and ^*DT*^*CCS*_*N2*_; and (2) a small positive bias was apparent for the ^*TIM*^*CCS*_*N2*_ [M+Na]^+^ data, which is notably different to the corresponding comparisons for protonated and deprotonated ions. Detailed assessment of individual compounds revealed that structural effects for some steroids may play an important role for comparability of measurements on different IM-MS instruments. For example, comparisons of ^*TW*^*CCS*_*N2*_ with ^*DT*^*CCS*_*N2*_ and for ^*TIM*^*CCS*_*N2*_ with ^*DT*^*CCS*_*N2*_ revealed that the ion with largest bias was [M–H]^−^ of estradiol diglucuronide (EDG) which was the only analyzed diglucuronide with similar bias of 6.8% and 6.6%, respectively. In addition, [M+Na]^+^ ion of boldenone undecylenate (BU) had a bias of 4.2% between ^*TIM*^*CCS*_*N2*_ and ^*DT*^*CCS*_*N2*_ and a bias of 2.3% between ^*TW*^*CCS*_*N2*_ and ^*DT*^*CCS*_*N2*_ data. Furthermore, results for [M+H]^+^ of androstanolone and androstanedione with a bias of −1.9%, −1.8% between ^*TIM*^*CCS*_*N2*_ and ^*DT*^*CCS*_*N2*_ and a bias of −2.7%, −2.5% between ^*TW*^*CCS*_*N2*_ and ^*DT*^*CCS*_*N2*_ indicate similar differences between DTIM-MS and the two other instruments. In a complementary comparison of ^*TW*^*CCS*_*N2*_ and ^*TIM*^*CCS*_*N2*_ data (^*TIM*^*CCS*_*N2*_ used as reference) the average absolute bias was found to be 0.66% ± 0.39% with a maximum of 1.81% and 95th percentile of 1.34%. Overall, fewer ions with extremely large biases were observed in this comparison than in the case of using ^*DT*^*CCS*_*N2*_ values as the reference.

**Figure 1:**
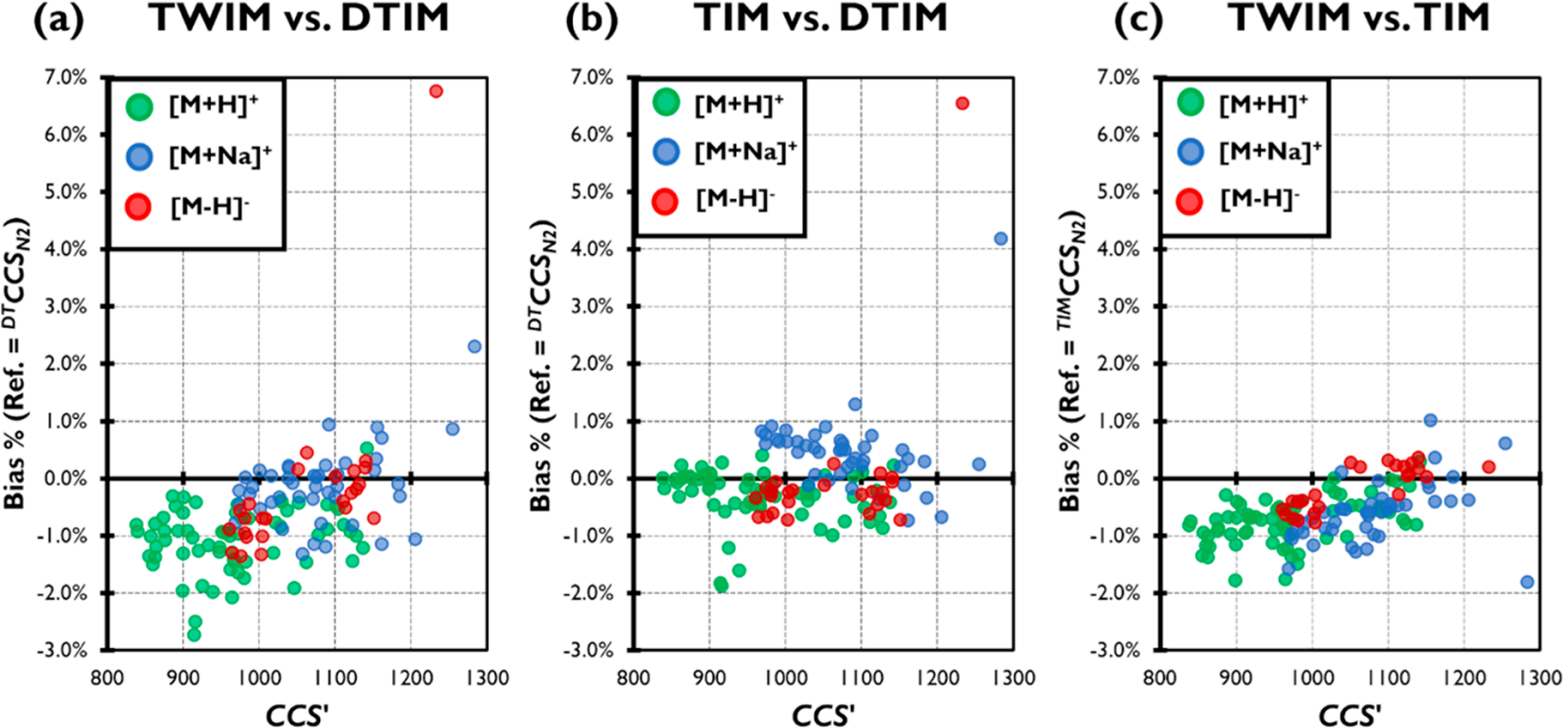
Bias (%, ref = ^*DT*^*CCS*_*N2*_) of (a) ^*TW*^*CCS*_*N2*_ and (b) ^*TIM*^*CCS*_*N2*_ as a function of *CCS′* for ion species [M+H]^+^, [M+Na]^+^, and [M–H]^−^. Panel (c) shows bias (%, ref = ^*TIM*^*CCS*_*N2*_) between ^*TW*^*CCS*_*N2*_ and ^*TIM*^*CCS*_*N2*_ as a function of *CCS′* for ion species [M+H]^+^, [M+Na]^+^, and [M–H]^−^. Pearson correlations (*r*) were calculated for the entire data sets.

To assess trends in the data sets with respect to the transport mechanism of IM, bias data was also plotted using the modified *CCS′* as an independent variable (*CCS*′ = *CCS* with μ being the reduced mass of the ion-gas pair and *z* the charge number). This comparison reflects the separation order and reveals a moderate positive correlation (Pearson correlation *r* = 0.535) for bias of individual ions’ ^*TW*^*CCS*_*N2*_ against ^*DT*^*CCS*_*N2*_ with respect to *CCS′*, while a weak positive correlation (*r* = 0.296) was observed in the corresponding comparison of ^*TIM*^*CCS*_*N2*_ and ^*DT*^*CCS*_*N2*_ (Figure 1). Assessment of the correlation of bias between ^*TW*^*CCS*_*N2*_ and ^*TIM*^*CCS*_*N2*_ with *CCS′* revealed broadly similar results (*r* = 0.550) as when ^*DT*^*CCS*_*N2*_ was used as the reference.

**Figure 2:**
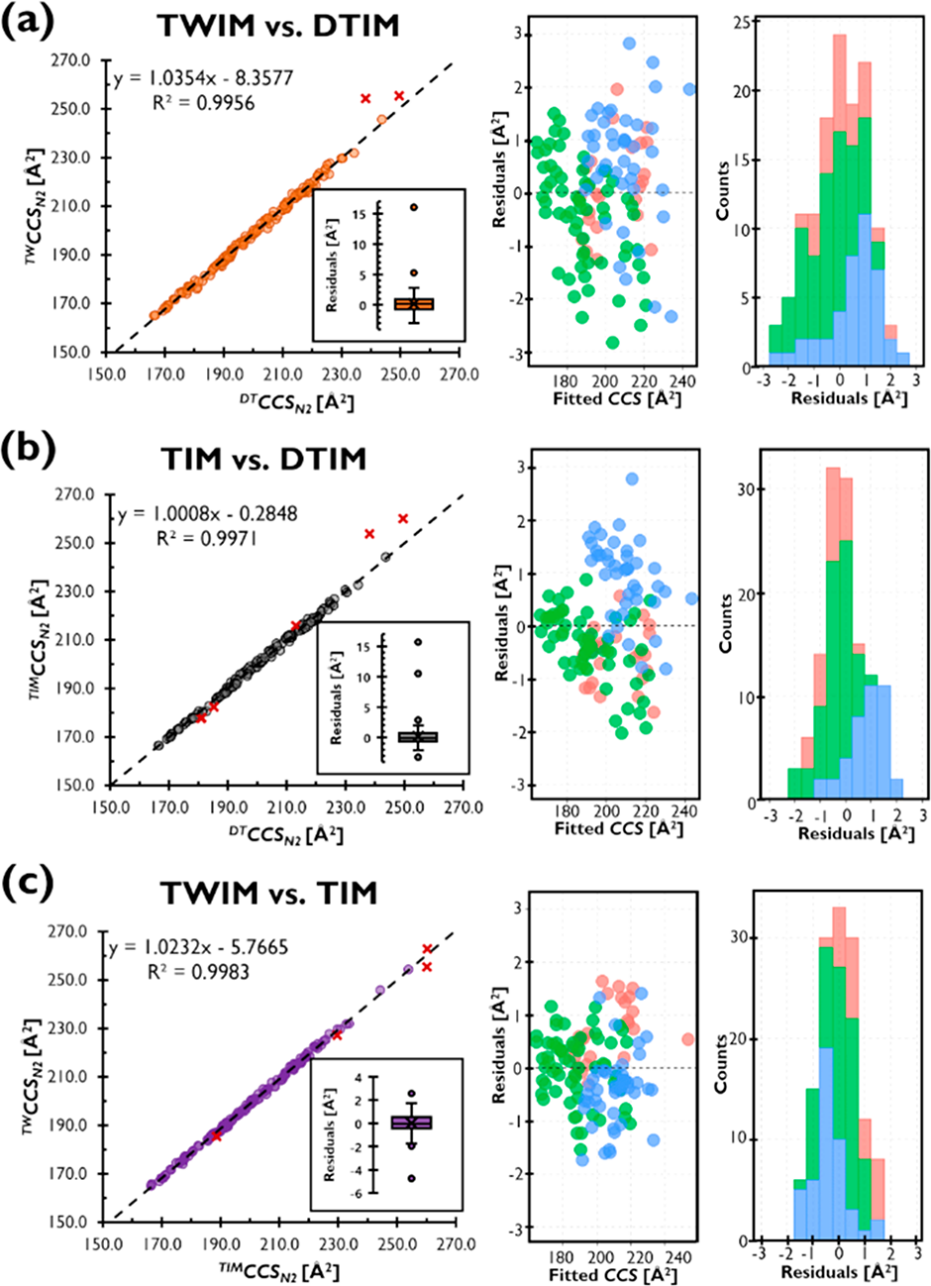
Linear regression models and residuals for comparisons of (a) ^*TW*^*CCS*_*N2*_ with ^*DT*^*CCS*_*N2*_, (b) ^*TIM*^*CCS*_*N2*_ with ^*DT*^*CCS*_*N2*_, and (c) ^*TW*^*CCS*_*N2*_ with ^*TIM*^*CCS*_*N2*_. Boxplots were used to determined outliers based on residuals and data points not included in the linear models are indicated as red crosses in the corresponding scatterplot. Residuals of outliers are not included in the corresponding scatterplots and histograms. Colors for ion species shown correspond to those in Figure 1.

Furthermore, linear regressions were used to investigate systematic bias and possible calibration-related contribution to these observations. A set of linear regressions comparing ^*DT*^*CCS*_*N2*_, ^*TIM*^*CCS*_*N2*_, and ^*TW*^*CCS*_*N2*_ was built for this purpose, and residuals were analyzed and ions with residuals outside of upper or lower whiskers were excluded from the linear models (Figure 2). The goodness of fit was excellent for all linear models with coefficients of determination (*R*^2^) ≥ 0.9956, but systematic differences were observed for the comparisons of ^*TW*^*CCS*_*N2*_ with ^*DT*^*CCS*_*N2*_ and for ^*TW*^*CCS*_*N2*_ with ^*TIM*^*CCS*_*N2*_. An intercept magnitude of <1 Å^2^ and a slope of 1.0008 in the linear model comparing ^*TIM*^*CCS*_*N2*_ and ^*DT*^*CCS*_*N2*_ were obtained, whereas intercepts of >5 Å^2^ in combination with steeper slopes (>1.02) remained for the linear models comparing ^*TW*^*CCS*_*N2*_ with ^*DT*^*CCS*_*N2*_ and ^*TIM*^*CCS*_*N2*_. It is noteworthy that ^*DT*^*CCS*_*N2*_ and ^*TIM*^*CCS*_*N2*_ are routinely calibrated with the same commercially available compound mixture (i.e., reference ions and reference values) established by Stow et al.,^23^ while ^*TW*^*CCS*_*N2*_ systems were calibrated using a different commercial calibrant mix. Our findings further evidence that an additional systematic difference is brought in by the external calibration strategy for TWIM-MS, here in the case of small molecules.

Residuals remaining within the whiskers of boxplots were all below ±3 Å^2^ and the majority within ±2.0 Å^2^ of fitted values. Moreover, the distribution of residuals for [M+Na]^+^ ions was observed to be positively shifted for the comparisons of both ^*TW*^*CCS*_*N2*_ with ^*DT*^*CCS*_*N2*_ and ^*TIM*^*CCS*_*N2*_ with ^*DT*^*CCS*_*N2*_ (blue histograms, Figure 2a,b), which is in agreement with the positive bias for those ions reported in Figure S2. Furthermore, even after removal of outliers, the spread of values for ^*TW*^*CCS*_*N2*_ vs ^*DT*^*CCS*_*N2*_ remained large in comparison to ^*TIM*^*CCS*_*N2*_ vs ^*DT*^*CCS*_*N2*_ or ^*TW*^*CCS*_*N2*_ vs ^*TIM*^*CCS*_*N2*_.

### Feasibility of Applying Single or Interplatform CCS Databases for Identity Confirmation of Steroids

**Figure 3:**
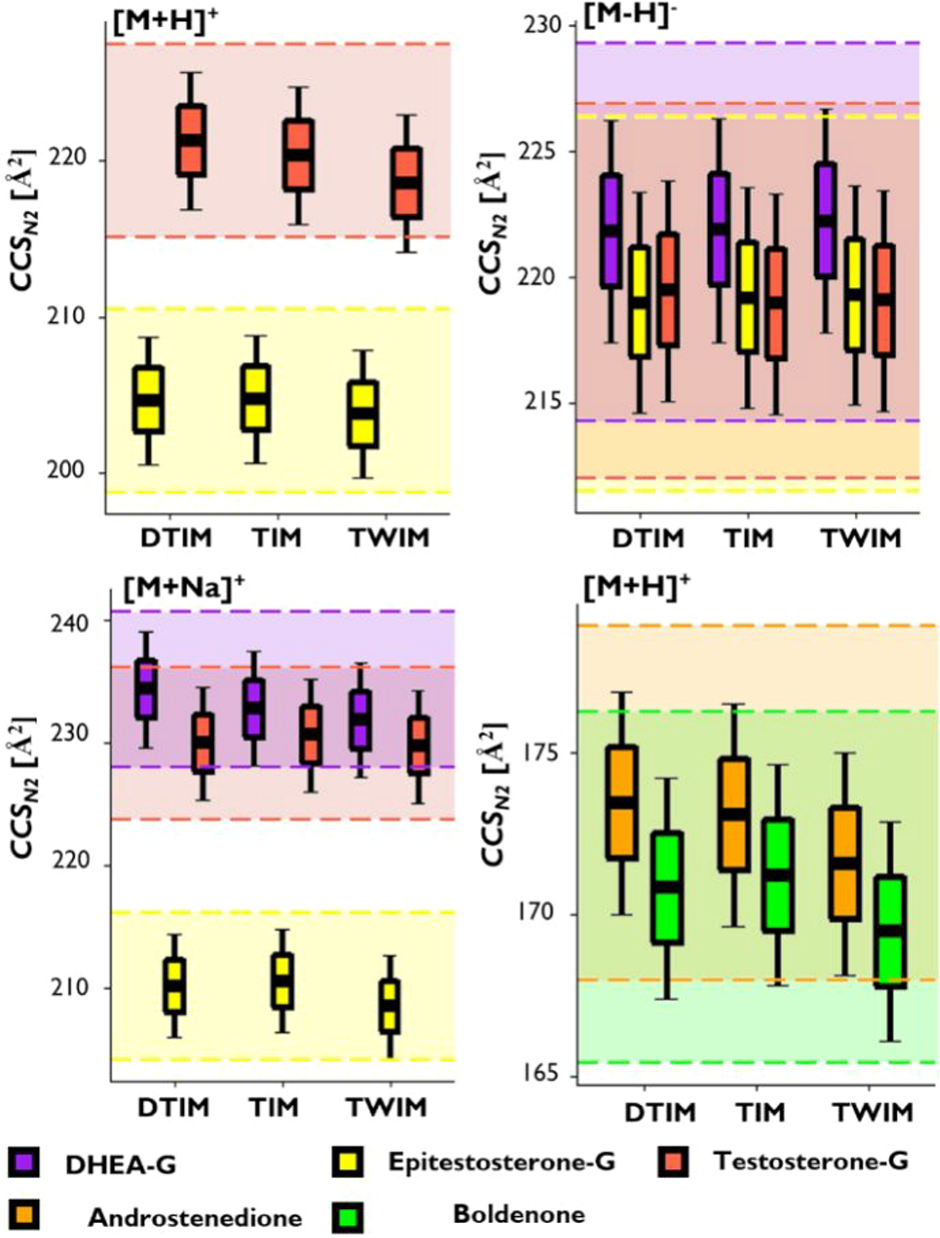
Comparison of ^*DT*^*CCS*_*N2*_, ^*TIM*^*CCS*_*N2*_, and ^*TWIM*^*CCS*_*N2*_ data for isomer examples assessed in this study. Boxes and whiskers indicate typically applied tolerance limits of ±1% and ±2%, respectively. Uncertainty estimate boundaries (coverage factor *k* = 1)^46^ calculated for ^*DT*^*CCS*_*N2*_ of respective ions are represented by the shaded areas bounded by dashed lines.

**Figure 4:**
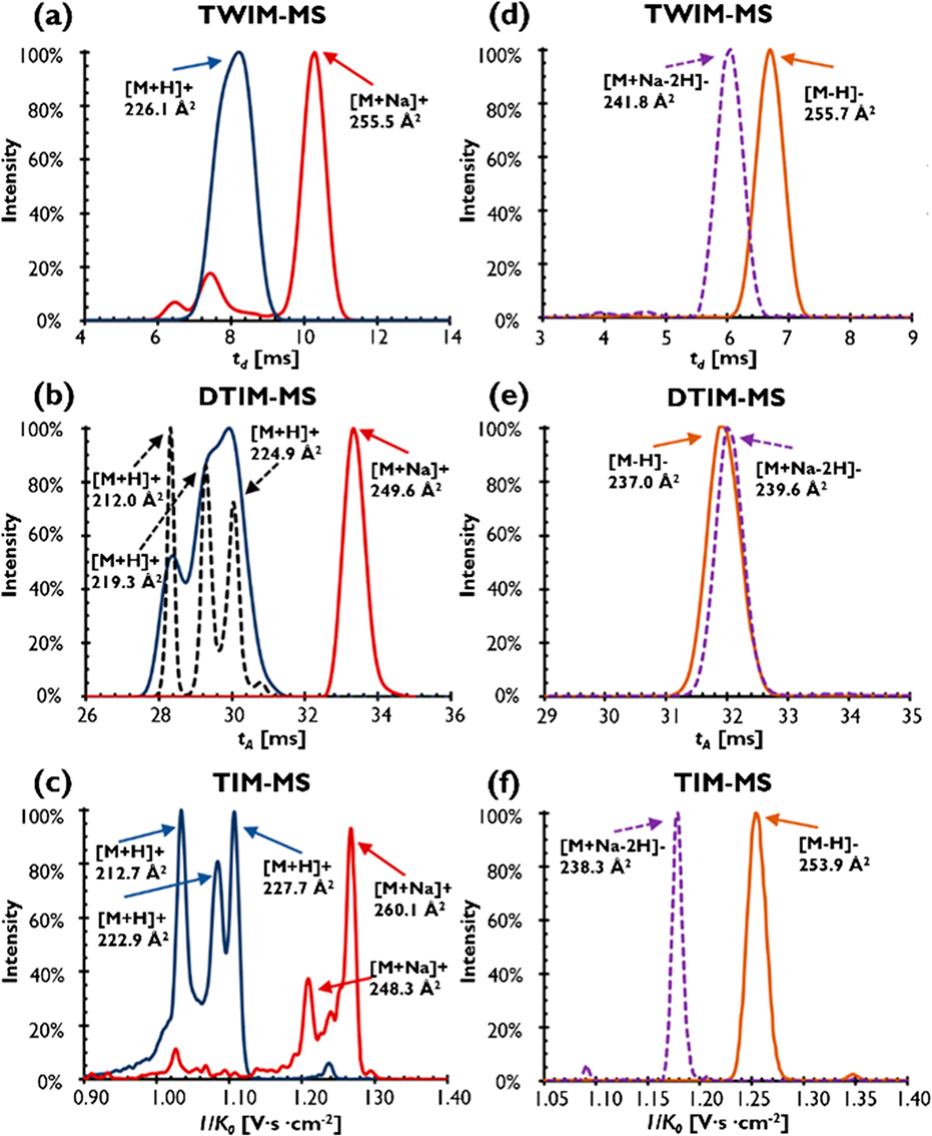
IM spectra for [M+H]^+^ and [M+Na]^+^ ions of boldenone undecylenate (BU) in (a) TWIM-MS, (b) DTIM-MS data, and (c) TIM-MS and arrival time spectra for [M–H]^−^ and [M+Na–2H]^−^ ions of estradiol diglucurondide (EDG) in (d) TWIM-MS, (e) DTIM-MS data, and (f) TIM-MS data. DTIM-MS Hrdm spectra (*R*_*p*_ ∼ 110) are represented by dashed black lines in panel (b). Intensity is normalized to the intensity of the largest peak in the IM spectrum.

The value of *CCS* from instrument-specific, crowd-sourced, or *in silico* databases remains a topic of great interest for a wide range of analytical applications where standards-free identity confirmation is demanded.^14,30,36^ Within our study, the generation of a large data set encompassing *CCS* values from the three major IM-MS instrument types enabled a critical view on the feasibility of either instrument-specific or interplatform *CCS* databases for the first time. Although several studies have demonstrated the excellent repeatability and minimal matrix effect for *CCS*_*N2*_ in discriminating different steroid isomers,^5,10^ uncertainty estimates for results and reference values must be considered when *CCS*_*N2*_ is employed as an identification parameter on a routine basis.^46^ However, reporting of accepted tolerance levels is usually pragmatic and oriented around observed interlaboratory reproducibility leading to precision estimates in the region of ±1% for DTIM-MS,^12^ while ±2% is often considered for TWIM-MS and TIM-MS applications.^14,29,45^ To assess the merits of these limits in a clear way, average *CCS*_*N2*_ for each instrument type along tolerances of ±1% (boxes) and ±2% (whiskers) for a series of isomeric ions from the new data sets are plotted in Figure 3. In addition to these thresholds, uncertainty estimates (*U*, coverage factor *k* = 1) for ^*DT*^*CCS*_*N2*_ are plotted to illustrate the challenges using *CCS*_*N2*_ for assigning the correct identity from several possible isomers within a given database. For the selected subset of isomers, all average *CCS*_*N2*_ fall into the uncertainty estimates for single-field ^*DT*^*CCS*_*N2*_ and similar trends regarding isomer differentiation were observed on all instruments. Except for [M+H]^+^ ions of testosterone glucuronide, epitestosterone glucuronide and [M+Na]^+^ ion of epitestosterone glucuronide, unambiguous differentiation of these isomers across platforms is already impossible when a ± 1% tolerance is accepted without additional use of other identification criteria such as retention time information. Furthermore, the systematic bias observed in the ^*TW*^*CCS*_*N2*_ data would influence false positive and false negative candidates if databases of other instrument classes are used as reference. While only representing a small subset of a particular class of small molecules, this result illustrates the necessity of further collaborative efforts to investigate the merits of consolidating external calibration ions, reference *CCS* values, and strategies for *CCS* determination as well as the establishment and implementation of reference materials with a view toward applying *CCS* as reliable parameter within standards-free identity confirmation workflows. In considering the physical interpretation of *CCS* data, it can be stated that the ability to unambiguously diagnose outliers as being representative of true differences in ion conformation is currently hampered. Within the present study, several outliers for ^*TIM*^*CCS*_*N2*_ and ^*TW*^*CCS*_*N2*_ were suspected due to the large differences between −2.7% and +6.8% to the reference DTIM-MS values. However, it is noteworthy that only sodiated boldenone undecylenate (BU) and deprotonated estradiol diglucuronide (EDG) are outside the uncertainty estimates (*k* = 1) for ^*DT*^*CCS*_*N2*_. While representing only a small fraction (<1.5%) of the present data set, these large differences between ^*TIM*^*CCS*_*N2*_, ^*TW*^*CCS*_*N2*_, and ^*DT*^*CCS*_*N2*_ are a clear issue for the broad applicability of *CCS* as an identification parameter across different IM-MS platforms. Figure 4a–c shows IM data for [M+H]^+^ and [M+Na]^+^ ions of a sterol ester (BU). The DTIM data for [M+H]^+^ of BU (4-bit multiplexing) revealed two partly separated peaks with ^*DT*^*CCS*_*N2*_ of ∼213 and ∼222 Å^2^ as well as an additional shoulder indicating multiple gas phase conformations that are partly separated by DTIM (Figure 4b, solid blue line). Although no separation was observed in the TWIM-MS data at least in part due to its lower IM resolution, a broad arrival time distribution was observed hinting toward the presence of additional unresolved conformers (Figure 4a). Use of TIM-MS allowed partial resolution of three peaks with ^*TIM*^*CCS*_*N2*_ of 212.7, 222.9, and 227.5 Å^2^ (Figure 4c) while recently introduced high-resolution demultiplexing (Hrdm)^51^ for DTIM-MS data revealed a qualitatively similar result (Figure 4b, dashed line). While only one major IM peak was observed for the BU [M+Na]^+^ ion using DTIM-MS or TWIM-MS, a complex IM spectrum was obtained on the TIM-MS instrument including a dominant larger conformation (^*TIM*^*CCS*_*N2*_ = 260.1 Å^2^) and a peak with a similar conformation as on the DTIM-MS with ^*TIM*^*CCS*_*N2*_ = 248.3 Å^2^. One explanation is that the high degree of flexibility of the fatty acid chain of BU supports the formation of multiple different stable gas-phase conformations regardless of the used analyzer. However, further theoretical calculations would be required to fully answer this question. These observations limit the use of a simple platform-independent application of *CCS* for identification purposes for such compounds. In the second example (Figure 4d–f), IM spectra of EDG [M–H]^−^ are investigated in more detail. For this compound, several additional ion species were observed including [2M-2H]^2–^ (*m*/*z* 311.1136 with ^*DT*^*CCS*_*N2*_ ∼ 301 Å^2^), a doubly charged dimer (623.2345 *m*/*z* with ^*DT*^*CCS*_*N2*_ ∼ 388 Å^2^), and the [M+Na-2H]^−^ ion. Interestingly, the [M–H]^−^ ion exhibited a more compact conformation in DTIM-MS compared to TIM-MS and TWIM-MS, whereas the *CCS*_*N2*_ for [M+Na-2H]^−^ was ∼240 Å^2^ across all data sets. The large *CCS* difference of >6.5% indicates that a different conformation of the [M–H]^−^ ion is sampled in DTIM-MS compared to the other instruments. However, this might be related to fundamental differences of the ion transport mechanism, the ion structure or due to the influence of ESI source conditions used on the different instrument classes. To fully elucidate such results would require measurements with the same ESI source and conditions applied on different IM-MS platforms to be performed, which was not feasible within the context of this study. Such examples finally underscore the value of carefully curated and instrument class-specific experimental *CCS* databases targeting specific analytical applications although the agreement of *CCS* values derived from different instrument classes is very good for most of the investigated ions. Furthermore, although high IM resolving power is clearly beneficial for overcoming some of the current limitations of IM-MS analysis for isomeric small molecules, the issues of external calibration and the influence of measurement conditions affecting ion conformation must be considered separately. Recently commercialized IM-technologies such as the Structures for Lossless Ion Manipulations (SLIM)-MS,^52^ cyclic ion mobility (cIM),^53^ and TIM-MS^54^ can be operated with *R*_p_ > 200, while HRdm for DTIM-MS allows a comparable increase via data processing.^51^ Thus, as the range of commercial IM-MS technologies with expanded resolution capabilities increases, external calibration will remain as a critical issue for derivation and comparison of *CCS* values.

## Conclusion

This study systematically investigated the comparability of ^*TW*^*CCS*_*N2*_, ^*TIM*^*CCS*_*N2*_ and ^*DT*^*CCS*_*N2*_ for the analysis of steroids and phase II metabolites for the first time. Most of the investigated ions fell within ±1% of reference ^*DT*^*CCS*_*N2*_ for TIM-MS and within ±2% for TWIM-MS. Additionally, 95% of the ^*TW*^*CCS*_*N2*_ values were found within ±1.34% of reported ^*TIM*^*CCS*_*N2*_ values despite a systematic negative and *CCS′*-dependent bias in ^*TW*^*CCS*_*N2*_ data compared to DTIM-MS and TIM-MS data.

Our findings also revealed the presence a calibration-dependent bias for TWIM-MS that is not apparent between DTIM-MS and TIM-MS data sets which are routinely calibrated with the same set of tune ions. While overall agreement was found to be good across all three platforms, these observed systematic differences for TWIM-MS hamper the applicability of *CCS* databases across different types of IM-MS technologies and increase the risk of false positive and false negative identifications.

Furthermore, by considering uncertainty estimates associated with ^*DT*^*CCS*_*N2*_ reference values, a new approach toward the unambiguous identification of outliers was presented. Only a small number of ^*TIM*^*CCS*_*N2*_ and ^*TW*^*CCS*_*N2*_ values (i.e., <1.5% of the ions) were found to be substantially different from ^*DT*^*CCS*_*N2*_ values and their uncertainty estimates. However, whether these experimental differences are due to the different IM-separation mechanisms or originating from the influence of different ESI parameters cannot be ascertained on a uniform basis.

From a broad analytical perspective, while the unavailability of true *CCS* values remains as the major hindrance in evaluating the use of *CCS* for identification workflows, harmonization of calibrant ions and their reference ^*DT*^*CCS*_*N2*_ employed for external instrument calibration demands for further investigation. Good analytical practices including validation of external calibration and better modeling of measurement uncertainty remain at the heart of IM-MS research if this technology is to make the transition from research into routine analytical laboratories.

To the best of our knowledge, this work encompasses the first and most comprehensive comparison of *CCS* values obtained from three major classes of IM-MS instruments for the analysis of steroids so far. While limited to steroids within the present study, these approaches can be viewed as a model that can be applied to a broader range of small molecule databases whereby any of the three instrument classes might be utilized and *CCS* values employed for supporting standards-free identity confirmation.

## Supporting Information Available

Additional experimental method information, description of external calibration approaches for IM-MS, additional data analysis, and visualization (PDF)
List of steroid mixtures and solvents used in this study (XLSX)
Complete interplatform *CCS*_*N2*_ database (XLSX)
