# Supplementary material for: Comparability of Steroid Collision Cross Sections Using Three Different IM-HRMS Technologies: An Interplatform Study
Source: J Am Soc Mass Spectrom. 2022 Sep 1;33(10):1951–9. doi: 10.1021/jasms.2c00196 (PMC9545150; doi:10.1021/jasms.2c00196)
Supplement: Supplementary file 1 — js2c00196_si_001.pdf [file js2c00196_si_001.pdf]

Supporting information for: Comparability of Steroid Collision Cross Sections Using Three Different IM-HRMS Technologies: An Interplatform Study

Max L. Feuerstein[1], Maykel Hernández-Mesa[2], Andrea Kiehne[3], Bruno Le Bizec[2], Stephan Hann[1], Gaud Dervilly[2], Tim Causon[1]*

----

[1] University of Natural Resources and Life Sciences, Vienna (BOKU), Department of Chemistry, Institute of Analytical Chemistry, Muthgasse 18, 1190 Vienna, Austria

[2] Oniris, INRAE, LABERCA, 44300 Nantes, France

[3] Bruker Daltonics GmbH & Co. KG, 28359 Bremen, Germany

*Corresponding author

Tel: +43 1 47654-77187

E-Mail: Tim.Causon@boku.ac.at

University of Natural Resources and Life Sciences, Vienna

Department of Chemistry

Institute of Analytical Chemistry

Muthgasse 18, Vienna, 1190

Austria

Table of Contents

External Calibration for IM-MS 3

Drift tube ion mobility mass spectrometry (DTIM-MS) 3

Traveling wave ion mobility-mass spectrometry (TWIM-MS) 4

Trapped ion mobility-mass spectrometry (TIM-MS) 5

(LC-)IM-QTOFMS Methods 6

Data acquisition 6

Data pre-treatment 8

Data processing 8

Additional Data Analysis and Visualization 10

Data analysis and visualization 10

Analytical performance of the individual IM-MS platforms 10

Comparison to published $^{DT}CCS_{N2}$ data 18

References 19

External Calibration for IM-MS

Drift tube ion mobility mass spectrometry (DTIM-MS)

In DTIMS, ion mobility (K, $m^2s^{-1}V^{-1}$) depends on the steady state drift velocity (v_d, ms^{-1}) and the strength of the applied electric field. In the case of a linear drift tube of length (L, m), an ion's mobility (Eq. 1) can be calculated from the drift time (t_d, s) and is often reported as a reduced mobility K_0 (Eq. 2) by taking into account normalized pressure and temperature with N representing the gas number density and N_0 the Loschmidt constant, p_0 and T_0 are NIST standard temperature and pressure, respectively.[1,2]

$$K = \frac{v_d}{E} = \frac{L}{t_d E} \quad (1)$$

$$K_0 = K \cdot \frac{N}{N_0} = K \cdot \frac{p}{p_0} \cdot \frac{T_0}{T} \quad (2)$$

Using the fundamental ion mobility equation under the assumption of zero field conditions, a collision cross section or collision integral (CCS, Å^2) can be derived from K_0 via Eq. 3:

$$CCS = \frac{3}{16}\sqrt{\frac{2\pi}{\mu k_B T}}\frac{ze}{N_0 K_0} \quad (3)$$

Hence, CCS also depends on the reduced mass (μ, kg) of the ion-gas pair, charge ($z \cdot e$, C), temperature (T, K), and the Boltzmann constant (k_B, JK^{-1})[1,2]. As experimentally determined arrival times t_A measured in DTIM-MS consist of the drift time t_d plus the time spent outside of the drift tube t_0, CCS calculation requires either an external calibration (secondary method) or measurements performed at multiple field strengths. In the latter case, arrival times are recorded at multiple voltages and drift times are corrected by the factor t_0 using a linear regression approach (Eq. 4).[3]

$$t_A = \frac{L^2}{K_0}\left(\frac{T_0}{p_0 T}\right)\left(\frac{P}{V}\right) + t_0 \quad (4)$$

In practice, the external calibration of DTIM-MS is achieved using an infusion of ions with known CCS to construct an external calibration curve with slope β and intercept t_{fix} at a single field strength (Eq. 5). Here, β and t_{fix} depend on applied conditions (e.g., drift gas, E, field strength, ion optics), m_g represents mass of the buffer gas and m_i the mass of the analyte ion[3].

$$t_A = \frac{\beta}{z}\left[\frac{m_i}{m_g + m_i}\right]^{1/2} CCS + t_{fix} \quad (5)$$

DTIMS or DTIM-MS operating as a primary method is used to establish reference values for external calibration. Excellent interlaboratory reproducibility (0.34%) and low interlaboratory bias (0.54%) compared to a reference instrument have been reported for DTIM-MS[3].

Traveling wave ion mobility-mass spectrometry (TWIM-MS)

TWIM transmission and separation relies on application of non-uniform fields and travelling potential waves which drag ions through the drift gas[4–6]. Typically, such TWIM-MS platforms provide IM resolving powers below ~40-60 depending on the instrument generation[6]. TWIM separation is a complex process in which ions of lower mobility can be overtaken by a potential wave resulting in a back-and-forth movement along the separation path. Due to the complex ion movement and related non-linear contributions to ion mobility, $^{TW}CCS_{N2}$ relies exclusively on external calibration which is (analogous to DTIM-MS; see Eq. 5) corrected by charge state and reduced mass to yield a modified *CCS* (*CCS'*, Eq. 6).[6–8]

$$CCS' = \frac{CCS\sqrt{\mu}}{z} \quad (6)$$

Experimentally determined arrival times are corrected to t_d' to account for non-IM-related contributions using a correction via the instrument dependent coefficient *C* and the ions' *m/z.*[6,7]

$$t'_d = t_A - \frac{C\sqrt{m/z}}{1000} \qquad (7)$$

Calibration coefficients *A* and *B* are then determined by fitting of logarithmic plot (Eq. 8) and $^{TW}CCS_{N2}$ of unknown ions is calculated using a power function (Eq. 9).[6,7]

$$ln\, CCS' = B \cdot ln\, t'_d + ln\, A \qquad (8)$$

$$CCS = A \cdot {t'_d}^B \cdot \frac{z}{\sqrt{\mu}} \qquad (9)$$

With TWIM, ions can experience high electric field strengths resulting in ion heating during ion injection and TWIM separation. This in turn leads to increasing vibrational energies and subsequent changes in ion conformation including isomerization or fragmentation[9]. The interlaboratory comparability for different types of TWIM-MS instruments is reported to be better than ±1.5% for the majority of investigated compounds in two interlaboratory comparisons[10,11]. However, this is only true when the same external calibration method and calibration ions are used. In contrast to DTIM-MS, the choice of calibrant ions is reported to have an impact impact in TWIM-MS calibration[7] and different calibration strategies including various calibrant ion mixtures have been reported[5,7,12]. Recent progress in development of universal calibration approaches aim at replacement of the currently used empirical power law function, but solving equations for TWIM ion motion is necessary for this purpose[12]. However, such calibration procedures are not currently used on a routine basis and a detailed discussion is out of scope for the present work.

Trapped ion mobility-mass spectrometry (TIM-MS)

In contrast to time-dispersive IM technologies, TIM-MS involves the use of a gas flow and a counteracting electrical field allowing accumulation (trapping) and selective release of ions. Ions are dragged through the TIM device using a gas flow, while an electromagnetic field gradient (EFG) is used to hold ions in a stationary position along with radio frequency (RF) potentials applied to achieve for radial confinement[13]. The dragging force of the gas flow with a defined gas velocity v_{gas} and the applied EFG counteract each other and ions are trapped along the EFG depending on their mobility[14]. By reducing the counteracting electric field strength (E_{trap}) ions are selectively released according to their mobility[15]. The separation occurs while ions traverse the plateau of the EFG[16] and ions are released in reverse order in comparison to DT or TWIM[4,17]. This results in high resolving powers (>100) using a relatively small device[17]. TIM-MS is typically externally calibrated using an empirical approach and ions with known K_0 are used (e.g., Agilent ESI-L tune mix). Due to the operation principle, different gas pressures along the TIM tunnel can be observed, but the difference in drag force is reported to be small[18]. Like in DTIM, reduced mobilities can be calculated using Eq. 10, where V_m is the applied voltage at time of release from TIM and *a* and *b* are empirical constants[14]:

$$K_0 = \frac{p}{p_0}\frac{T_0}{T}\frac{v_{gas}}{E_{trap}} \approx a + \frac{b}{V_m} \qquad (10)$$

With this approach, $^{DT}CCS_{N2}$ of Agilent tune mix ions were reproduced with excellent agreement (95th percentile ~1%). However, a systematic error can be observed when high TIM ramp rates are used as the constants *a* and *b* depend on the operation settings, but the exact relationship remains unknown. Alternatively, a calibration based on solving the Boltzmann transport equation was recently reported[14]. However, this is not currently applied on a routine basis and detailed discussion is out of scope of the present work.

(LC-)IM-QTOFMS Methods

Data acquisition

Chromatographic separation was performed using an Acquity UPLC BEH C18 column (2.1 mm × 100 mm, 1.7 µm particle size, Waters) equipped with an in-line filter kit (0.2 µm, Waters). For all experiments, the mobile phase comprised 0.1% (v/v) formic acid (A) and 0.1% (v/v) formic acid in acetonitrile (B). The following gradients were applied for hydrophilic (Gradient 1; Mixes 1-5, 7, 8 & 10) and hydrophobic (Gradient 2; Mixes 6 & 9) mixtures of standards with a column temperature of 50°C, a flow rate of 550 µL/min (DTIM-MS) or 600 µL/min (TIM-MS) and an injection volume of 2.5 µL (DTIM-MS) or 5 µL (TIM-MS). Gradient programs are shown in **Table S1**.

Table S1. LC gradient conditions used for separation of steroid mixtures and samples

LC Gradient 1			LC Gradient 2		
**Time [min]**	**% A**	**% B**	**Time [min]**	**% A**	**% B**
0.0	95	5	0.0	50	50
0.3	95	5	2.0	50	50
9.6	57	43	9.6	10	90
13.5	1	99	13.5	1	99
15.5	1	99	15.5	1	99
16.0	95	5	16.0	50	50
19.5	95	5	19.5	50	50

Additional measurements for assessment of long-term stability of $^{DT}CCS_{N2}$ were made using a Phenomenex Kinetex C18 column (4.6 mm × 50 mm, 2.6 µm particle size) due to unavailability of the initially used column.

For DTIM-MS measurements, the following ESI settings were used on an Agilent 6560 IM-QTOFMS (Agilent Technologies, Santa Clara, CA) equipped with a Dual AJS ESI Ion Source: gas temperature was set to 225 °C and a gas flow of 8 L/min was used. Nebulizer was operated at 30 psi, sheath gas temperature and flow were set to 350°C and 12 L/min, the capillary voltage was 3500 V, and the nozzle voltage was set to 500 V. $^{DT}CCS_{N2}$ was determined using stepped-field and single-field methods in both polarities. The mass range was set to 50-1700 *m/z* and measurements were performed using the 2 GHz Extended Dynamic Range setting. This instrument platform can be used with maximum IM resolving powers of ~70[19] and was achieved a resolving power of 50-60 in the present work.

For direct infusion measurements, a syringe pump was used with flow rates of 1.8 mL/h and 1.2 mL/h for analyzing standard mixtures using stepped-field and single-field methods, respectively.

The DTIM-MS was operated with drift gas pressure of 3.95 Torr and a temperature of 299.5 K. For stepped-field acquisition, the total runtime was 3.5 min using time segments with different drift field strengths. The maximum drift time was set to 90 ms and 10 IM transients were summed for each frame. Trap fill time was 10000 µs and trap release time was set to 150 µs. IM drift tube exit voltage was set constant to 224 V, while drift tube entrance voltage was varied from 1074 V to 1674 V in 100 V steps (yielding 10.9, 12.1, 13.4, 14.7, 16.0, 17.2 and 18.5 V/cm), corresponding to E/N values between 8.56 Td and 14.53 Td. The rear funnel entrance was 217.5 V and rear funnel exit was 45 V.

For single-field calibration applied to LC-DTIM-MS measurements, an Agilent 1290 Infinity II UHPLC system was used for online separation of compounds. Analogous acquisition settings were applied except that a fixed drift tube entrance voltage of 1574 V (17.2 V/cm) and maximum drift time of 50 ms were used. Standard operation measurements were performed using an “all ions” fragmentation approach (DIA) using alternating high- and low energy frames with 20 V and 0 V collision energy. For 4-bit multiplexing (4m), 1.2 frames per second were recorded with 16 IM transients per frame. The trap fill time was 1250 µs and trap release time was 150 µs. External calibration files for all measurements were recorded for 30 s using the same settings prior to sample analysis (Agilent ESI-L G1969-85000). Tune files underwent the same data pre-treatment (i.e., demultiplexing and smoothing) as the sample datafiles and were subsequently used for external calibration of datafiles using IM-MS Browser 10.0.

All TIM-MS measurements were performed using a timsTOF pro (Bruker Daltonics, Bremen, Germany) hyphenated to an Elute UHPLC using an ESI source. Ion transfer was optimized for 50-1000 *m*/*z* and full scan spectra were recorded in the mass range of 20-1300 *m*/*z* in positive and negative polarity with 4 Hz spectra rate. Ion source capillary voltage was 4.5 kV, nebulizer gas pressure was 2.5 bar and drying gas flow was 10.0 L/min using a temperature of 250°C. Nitrogen was used as drying gas and nebulizer gas. TIM ion charge control was set to $5 \cdot 10^6$ and ion mobility was scanned from 0.45 to 1.45 V/cm^2 with accumulation and ramp times of 100 ms each. Furthermore, Funnel 1 and 3 RFs were set to 200 Vpp, Mutipole RF was 60 Vpp and the collision RF was set to 450 Vpp.
The TOFMS was calibrated using 10 mM sodium formate and a 7th order high-performance calibration. In addition to the external calibration, each sample was automatically post-run calibrated by injecting a 1:1 mixture of both calibrants at the beginning of each measurement.

In the case of TWIM-MS, the established single laboratory database is based on measurements performed using a Waters Synapt G2-S HDMS instrument, while interlaboratory reference values were determined as average ${}^{TW}CCS_{N2}$ from four Waters TWIM-MS systems (Synapt G2-S and Synapt G2-Si Q-IM-TOF-MS and two Vion IM-QTOF-MS instruments). In both studies external CCS calibration using Waters Major Mix was applied. For creation of **Figure 4**, single TWIM-MS measurements were performed using the same Synapt G2-S HDMS instrument from Waters (Manchester, UK) and settings as described previously[20].

Data pre-treatment

Multiplexed (LC-)DTIM-MS data was demultiplexed using PNNL Preprocessor 3.0 (2021.04.21) using following settings: "Step 2 (a): Multiplexed Data: Demux, Smooth, Spike Rem." and "Step 3: Saturation Repair" were ticked. Data was smoothed using Moving average filters with a step-size of 3 for LC and IM dimensions, "Signal Intensity Lower Threshold" was set to 20 counts, spike removal was applied with a minimum of one adjacent data point per dimension required and saturated peaks were repaired above an abundance level of 40%.

Prior to application of High Resolution Demultiplexing (HRdm), the same datafiles acquired in 4-bit multiplexing were processed with PNNL Preprocessor 4.0 (2021.10.27) and using the same settings as for the standard multiplexing, except that each drift bin was interpolated resulting in three drift bins.

Data processing

All DTIM-MS files were CCS calibrated using calibration coefficients from a suitable calibration file determined in IM-MS Browser 10.0. Replicates of data files (.d-format) were either evaluated in batches using Agilent Mass Profiler 10.0 or, in the case of stepped-field acquisition, were evaluated with Agilent IM-MS Browser 10.0.

To increase the apparent resolving power of DTIM-MS data, HRdm 2.0 software was additionally used to process the 4-bit multiplexed datafiles. Interpolated files obtained from PNNL pre-processor were used as input files and data was processed using following settings: HR processing level was set to "low", *m/z* width multiplier was set to 6 and IF multiplier setting of 1 was used resulting in an IM resolving power of ~110 for boldenone undecylenate $[M+H]^+$.

The following settings were used for feature finding (peak picking) in Mass Profiler 10.0: abundance was measured as maximum ion volume and charge state was limited to 1-2. For alignment and normalization, the retention time tolerance was set to ± 0.3 min, drift time tolerance was ±1%, the acceptable mass tolerance was set to ± (15.0 ppm + 2.0 mDa) and all

features with a Q-score ≤ 70.0 were rejected. Feature results were exported as .csv files and were further used for data analysis using Microsoft Excel and R.

For stepped-field data evaluation, the fourth time segment was selected in IM-MS Browser 10.0, and frames were summed. The isotopic envelope of a target ion (at least two ions were required) was selected and “Calculate CCS (Multi field)” was used. Result tables were exported as .csv files and further statistical analysis was performed.

All TIM-MS files were analyzed using Bruker TASQ (2021) software. After automatic recalibration, features were detected (peak picking) based on accurate mass and retention time. $^{TIM}CCS_{N2}$ was determined automatically in the TASQ software after automated integration of high-resolution EICs using a trace width of ±3 mDa and a retention time window of 0.5 min for peak detection. Results were averaged across three replicates. Ion quality was determined based on the principal ion only. The classic chromatogram peak finder mode was used, and ion signals were determined based on the signal height using 250 counts as minimum peak area and 50 counts as minimum intensity threshold. LC peaks were smoothed and de-noised using a Gaussian peak model and the minimum signal-to-noise ratio was 3 along with minimum 5 data points per peak and chromatogram slices of 2.0 min were used. Extracted ion mobilograms (EIMs) were smoothed using a Gaussian peak model and only mobilograms within a bin size of 0.2 Vs cm^{-2} were created.

For creation of **Figure 4**, TWIM-MS and TIM-MS datafiles were additionally extracted using MS-DIAL software (MS-DIAL 4.80 along with IBF-converter software). Data was extracted using following settings: MS tolerances were set to 0.01 Da and a mass range of 50-1200 Da was used with a maximum charge state of 2. Data was smoothed using a linear-weighted moving average filter with a box width of 3, the minimum peak height was set to 3000 counts and minimum required peak width was set to 5 datapoints. The retention time used for peak picking in the IM-dimension was set to 0.2 min and an alignment tolerance of 0.01 $Vs{\cdot}cm^{-2}$ was used for TIM-MS data. The same settings were used for TWIM-MS data, but 0.02 ms was used as mobility tolerance. IM spectra were extracted as .csv from single files and data was replotted in Excel.

Additional Data Analysis and Visualization

Data analysis and visualization

In addition to Microsoft Excel, R (4.1.2)[21] language together with RStudio (2021.9.1.372)[22] was used for creating graphs and analyzing data. The following packages were used for this purpose: dplyr[23], ggplot2[24], ggally[25], ggpubr[26] and ggbreak[27]. R base and dplyr were used to re-arrange data tables. Functions from R base were used for basic data analysis (e.g., creating linear models). Ggplot2, ggally and ggpubr were used to create raw plots, (e.g., correlation matrix, violin plots or residual plots and ggbreak was used to insert axis breaks into violin plots).

Analytical performance of the individual IM-MS platforms

Precision under repeatability conditions for measurement of ${}^{DT}CCS_{N2}$ was excellent for triplicate measurements. An average RSD of 0.14%±0.12% was obtained using the stepped-field method and the maximum observed RSD of 0.88% for the sodium adduct of estradiol 17-glucuronide was significantly higher than the second highest value (RSD = 0.57%). The average RSDs for the employed secondary methods (i.e., DTIM-MS single-field) were 0.03%±0.02% for standard operation and 0.02%±0.01% for 4m operation. Negligible differences between ${}^{DT}CCS_{N2}$ values determined with 4m and standard operations were reported (average 0.19%±0.17%). The difference between the stepped-field dataset and individual single-field methods was 0.41%±0.22% in standard mode and 0.33%±0.21% using 4m mode, which is comparable to previously reported results using the same type of instrument[3,28–30].

To investigate matrix effects, steroids were spiked into urine samples and ${}^{DT}CCS_{N2}$ in urine and standards were compared. No substantial matrix effects for ${}^{DT}CCS_{N2}$ in LC-IM-MS were observed for the investigated 69 ions in four urine samples from different animals for which differing matrix complexity has been reported previously[31]. The absolute difference between ${}^{DT}CCS_{N2}$ values in standards and spiked urine was 0.14%±0.12% in adult bovine (max. = 0.65%, 95th percentile = 0.34%) and 0.12%±0.09% in calf urine (max. = 0.48%, 95th percentile = 0.28%).

The long-term applicability of ${}^{DT}CCS_{N2}$ for this application was evaluated remeasuring samples in triplicate 9 months after initial measurements in 4m mode. This revealed an average absolute difference of 0.17%±0.09% with a maximum absolute difference of 0.44% for a total of 66 for $[M+H]^+$ and 25 $[M-H]^-$ species. Our new dataset was also compared to recently published ${}^{DT}CCS_{N2}$ values for steroids analyzed with the same type of DTIM-MS instrument. Davis and co-workers published a ${}^{DT}CCS_{N2}$ database containing 22 $[M-H]^-$ corresponding to phase II metabolites of steroid doping agents[28], while Velosa et al. published ${}^{DT}CCS_{N2}$ a

dataset containing 48 ions corresponding to 26 anabolic steroids[29]. Overall, 24 ions of 16 steroids and 6 ions of steroid phase II metabolites that were measured in these previous studies were also analyzed in our work. The average absolute difference for single field-calibrated $^{DT}CCS_{N2}$ was 0.23% for positive mode data and 0.33% for deprotonated ions of phase II steroid metabolites (max. = 0.66%) illustrating the excellent interlaboratory agreement using DTIM-MS instruments of the same class (see **Table S2**).

Precision under repeatability conditions for measurement of $^{TIM}CCS_{N2}$ determination was also excellent with an average RSD of 0.13%±0.10% for the 142 investigated ions (n=3-6) and a maximum RSD of 0.72% for $[M+Na]^+$ ion of testosterone glucuronide. Overall, 95% of the investigated $^{TIM}CCS_{N2}$ values yielded an RSD ≤0.32%.

Matrix effects were evaluated using the same approach as for DTIM-MS and no substantial matrix effects were observed. The absolute difference between $^{TIM}CCS_{N2}$ in bovine urine was 0.13%±0.14% (95th percentile = 0.36%) in positive mode, while the absolute average difference was 0.12%±0.09% (95th percentile = 0.31%) in negative mode. Overall, the maximum observed difference was 1.00% for protonated 14α-hydroxytestosterone.

A series of our previous studies focused on steroid analysis with TWIM-MS and has encompassed a wide range of analytical performance metrics. The long-term applicability of $^{TW}CCS_{N2}$ has been previously accessed for a single Synapt G2-S instrument revealing that $^{TW}CCS_{N2}$ values for 99.8% of the studied steroid ions were found to be within ≤1.3% over 4 months and only negligible effects of urine matrix were reported[31]. Finally, an interlaboratory $^{TW}CCS_{N2}$ database based on four instruments from three different TWIM-MS generations (Waters Synapt G2-S HDMSE, Synapt G2-Si, and two Vion IM-MS systems) revealed some differences in $^{TW}CCS_{N2}$, but 96.8% of the reported measurements were within ±1.5% of the interlaboratory reference $^{TW}CCS_{N2}$ values[10].

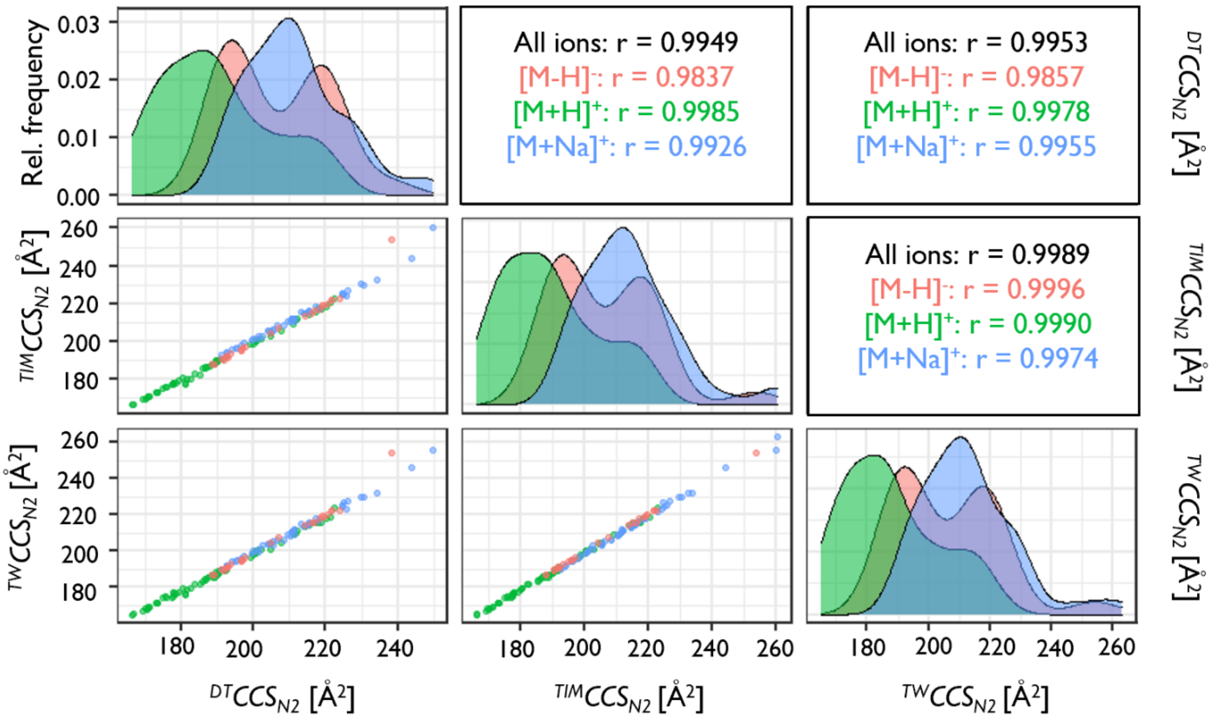

Figure S1. Correlation plots, *CCS* data distribution (fitted frequency density plots) and Pearson correlation coefficients of $^{DT}CCS_{N2}$, $^{TIM}CCS_{N2}$, and $^{TW}CCS_{N2}$ of $[M-H]^-$, (red) $[M+H]^+$ (green) and $[M+Na]^+$ (blue) ions of steroids and phase II steroid metabolites.

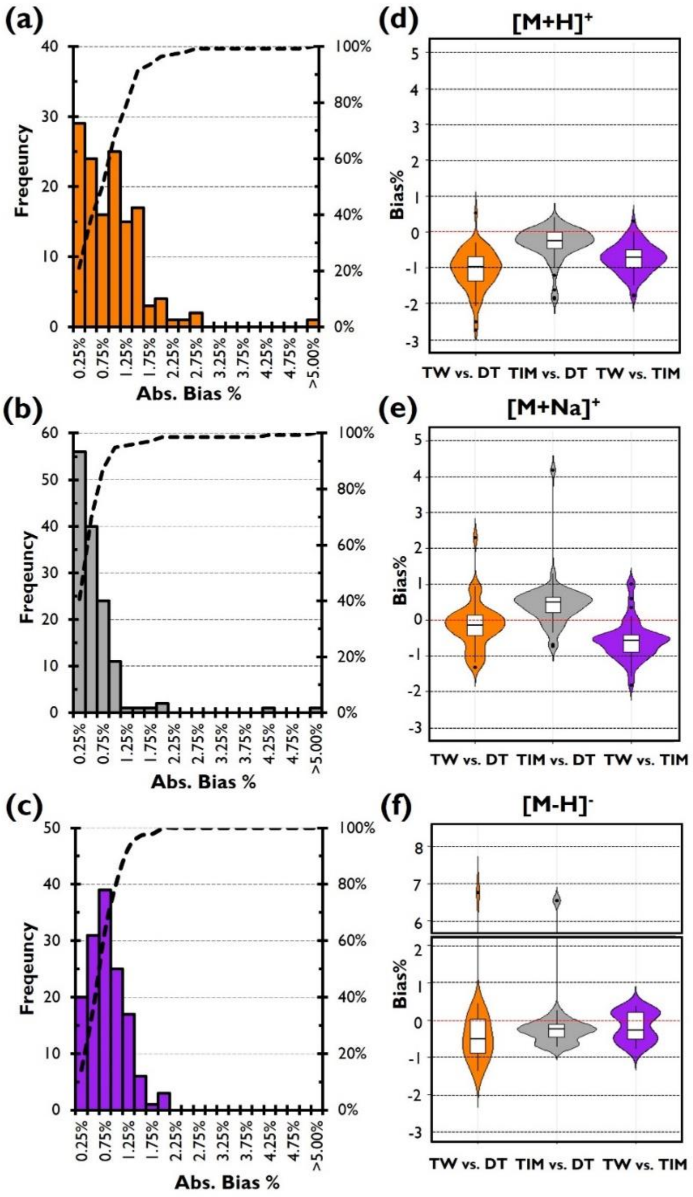

Figure S2. Histograms representing the corresponding absolute bias distribution for (a) $^{TW}CCS_{N2}$ and (b) $^{TIM}CCS_{N2}$ datasets compared to $^{DT}CCS_{N2}$; and (c) $^{TW}CCS_{N2}$ compared to $^{TIM}CCS_{N2}$. Violin plots representing the bias distribution of all three comparisons for (d) $[M+H]^+$, (e) $[M+Na]^+$ and (f) $[M-H]^-$ ions.

A bimodal distribution of bias for the $[M-H]^-$ ions is apparent (purple series, **Figure S2f**), and can be explained due to the subsets of investigated ions in negative mode (i.e., steroid sulfates and steroid glucuronides) forming two separate clusters.

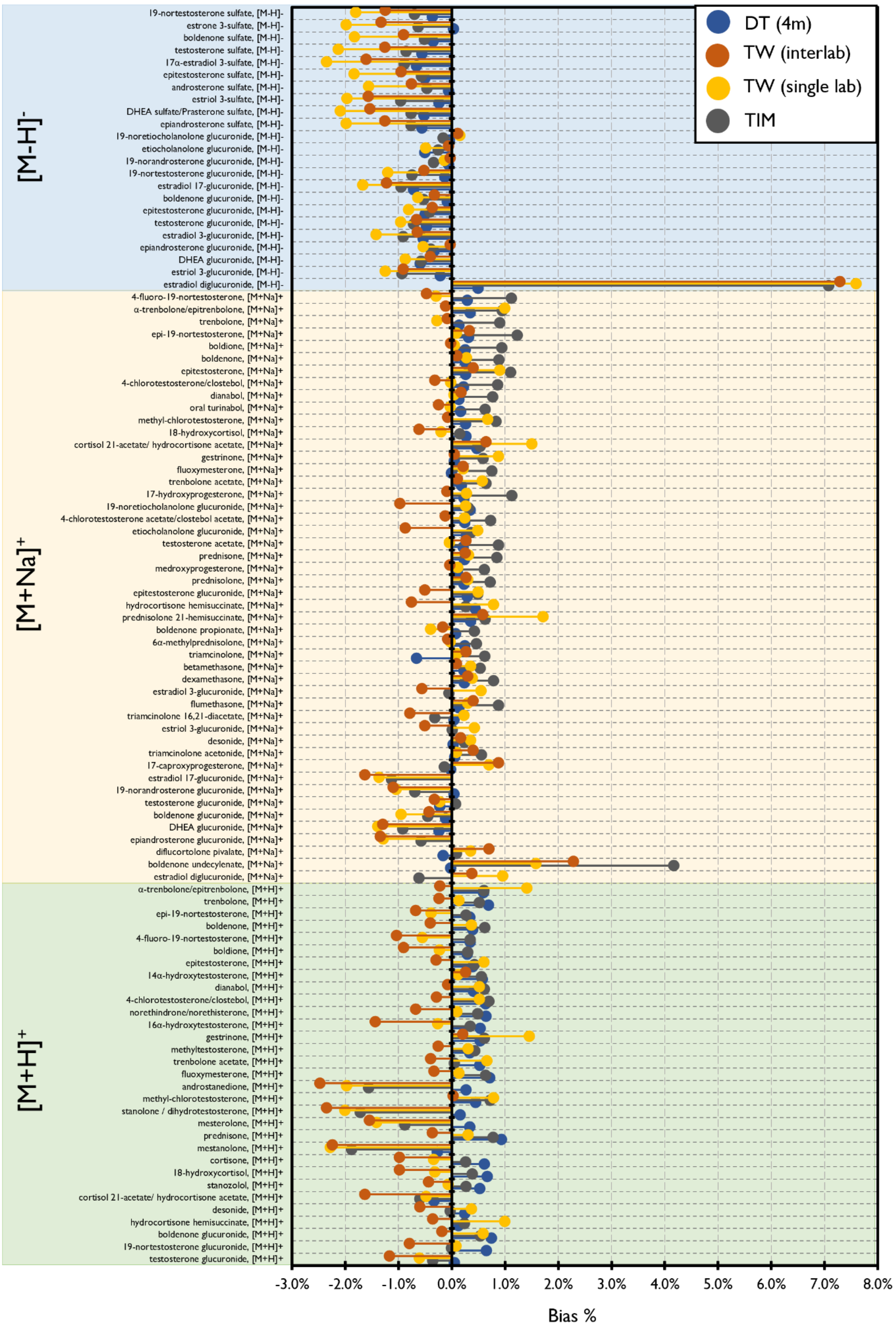

Figure S3. Bias of calibrated results from DTIM-MS (blue), TIM-MS (grey) and TWIM-MS against stepped-field ${}^{DT}CCS_{N2}$ as reference. For completeness, single laboratory (yellow) and interlaboratory values for ${}^{TW}CCS_{N2}$ (orange) are both included.

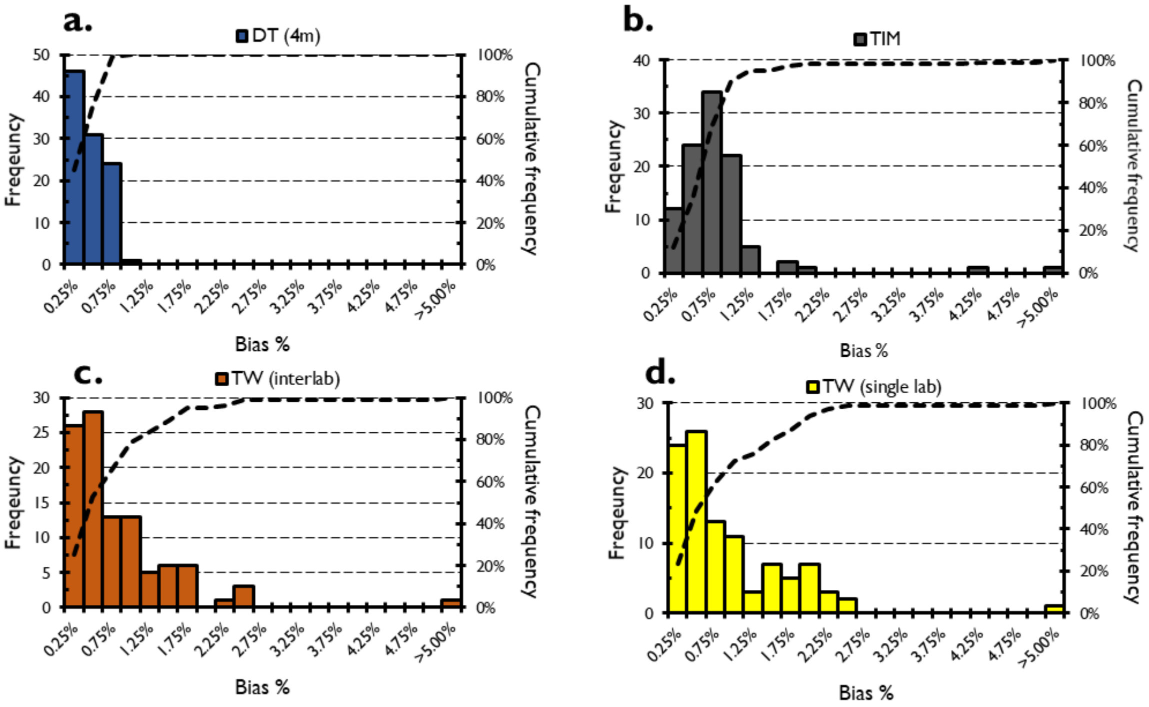

e.

Bias% Percentile	DT (4m)	TIM	TW (interlab)	TW (single lab)
**95th**	0.67%	1.22%	1.64%	2.01%
**90th**	0.63%	0.96%	1.53%	1.83%

Figure S4. Absolute bias of calibrated methods on DTIM-MS (a), TIM-MS (b) and TWIM-MS (c) using stepped field $^{DT}CCS_{N2}$ as reference. For completeness, single laboratory $^{TW}CCS_{N2}$ (d, yellow) are also shown. Panel (e) shows 90th and 95th percentile of absolute bias for all datasets.

Figure S4 shows the absolute bias of secondary calibrated IM-MS methods against stepped-field $^{DT}CCS_{N2}$ as reference:

$$\text{Bias [\%]} = \frac{^{Exp.}CCS_{N2} - {^{Ref}CCS_{N2}}}{^{Ref}CCS_{N2}} \quad (11)$$

The corresponding bias for each ion sorted by ion species and *CCS'* is shown in **Figure S3.** Absolute bias was smallest between single-field calibrated DTIM-MS recorded on the same instrument with an average absolute bias of 0.31±0.22% and a narrow bias distribution (95th percentile 0.67%). This was followed by TIM-MS, which had a higher average absolute bias of 0.71±0.80% and 95th percentile of 1.22%. A similar average absolute bias was observed for single laboratory and interlaboratory $^{TW}CCS_{N2}$ with average absolute bias of 0.70±0.87% and 0.81±0.93%, respectively. 95th percentiles were 1.64% and 2.01%, respectively.

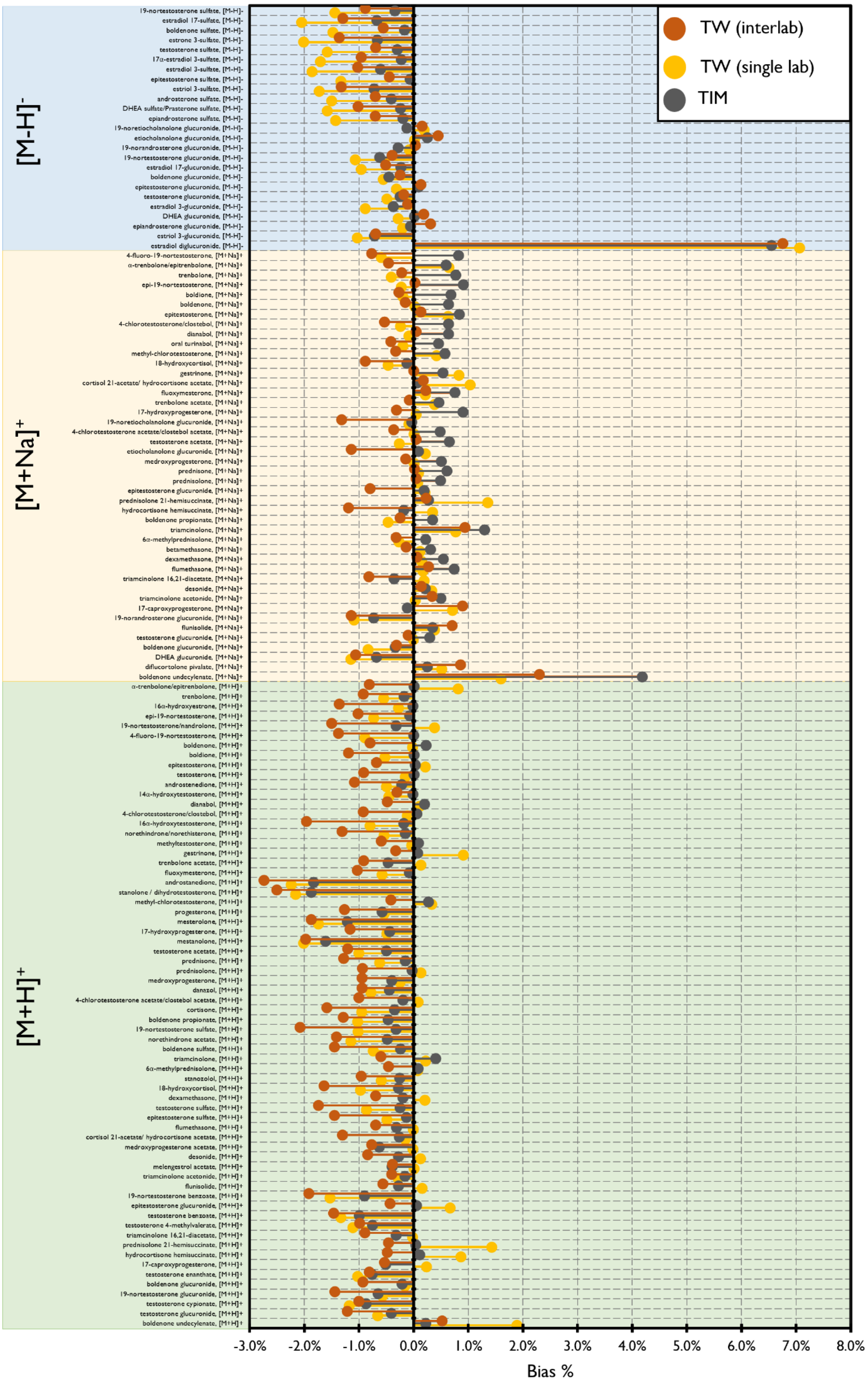

Figure S5. Bias of calibrated methods TIM-MS (grey) and TWIM-MS using single-field $^{DT}CCS_{N2}$ as reference. For completeness, single laboratory (yellow) and interlaboratory values for $^{TW}CCS_{N2}$ (orange) are both included.

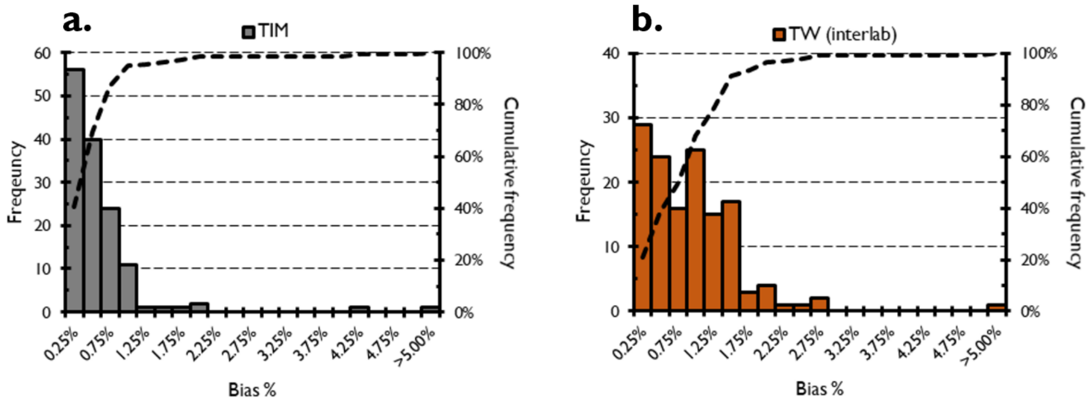

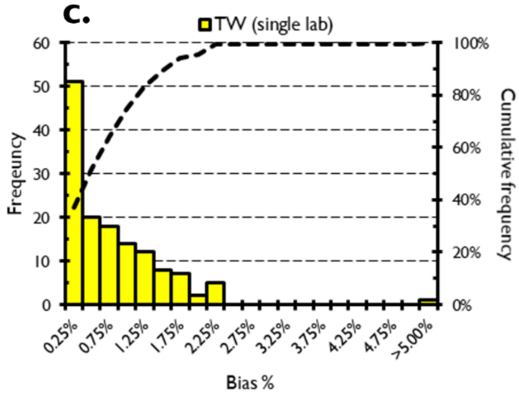

d.

Bias % Percentile	TIM	TW (interlab)	TW (single lab)
95th	1.03%	1.92%	1.87%
90th	0.78%	1.45%	1.54%

Figure S6. Absolute bias of secondary calibrated methods on (a) TIM-MS and (b) TWIM-MS using single-field $^{DT}CCS_{N2}$ as reference. For completeness, (c) single laboratory values for $^{TW}CCS_{N2}$ are also shown. Panel (d) shows 90th and 95th percentile of absolute bias for all datasets.

Bias for each ion sorted by ion species and *CCS'* is shown in **Figure S5. Figure S6** illustrates the absolute bias of secondary TIM-MS and TWIM-MS using single field $^{DT}CCS_{N2}$ as reference values. Absolute bias was smallest for TIM-MS with an absolute bias of 0.47±0.70% and 95% of the values within 1.03%. The average absolute bias observed for single laboratory and interlaboratory $^{TW}CCS_{N2}$ were 0.82±0.76% and 0.68±0.79% with 95th percentiles being 1.92% and 1.87%, respectively.

Comparison to published $^{DT}CCS_{N2}$ data

Comparisons of $^{DT}CCS_{N2}$ data determined on the same class of DTIM-MS instrument in two recent studies revealed good agreement for a small set of both steroids and steroid phase II metabolites. A small systematic bias (≤0.5%) for $[M-H]^-$ of 6 steroid phase II metabolites in one study[28] while a larger set of 24 $[M+H]^+$ and $[M+Na]^+$ ions revealed a bias of ≤0.7%[29] (**Table S2**).

Table S2. Comparison of published $^{DT}CCS_{N2}$ data with experimental data of this study. All data are single-field $^{DT}CCS_{N2}$ values from the Agilent DTIM-MS instrument.

Name	Formula	Ion	*m/z*	Ref. $^{DT}CCS_{N2}$ [Å²]	Meas. $^{DT}CCS_{N2}$ [Å²]	Bias %
**Boldenone glucuronide**	C25H34O8	$[M-H]^-$	461.2181	217.4[28]	218.22	0.4%
**Nandrolone glucuronide**	C24H34O8	$[M-H]^-$	449.2181	215.4[28]	216.32	0.4%
**Epitestosterone sulfate**	C19H28O5S	$[M-H]^-$	367.1585	193.0[28]	193.48	0.2%
**Prasterone sulfate (3β)**	C19H28O5S	$[M-H]^-$	367.1585	196.4[28]	196.81	0.2%
**Epiandrosterone sulfate**	C19H30O5S	$[M-H]^-$	369.1741	197.4[28]	197.73	0.2%
**Androsterone sulfate**	C19H30O5S	$[M-H]^-$	369.1741	195.7[28]	196.75	0.5%
**Trenbolone**	C18H22O2	$[M+H]^+$	271.1689	166.6[29]	166.7	0.1%
**Trenbolone**	C18H22O2	$[M+Na]^+$	293.1518	193.0[29]	192.6	-0.2%
**Nandrolone**	C18H26O2	$[M+H]^+$	275.2011	170.2[29]	170.6	0.2%
**Boldione**	C19H24O2	$[M+H]^+$	285.1855	170.8[29]	171.2	0.2%
**Boldione**	C19H24O2	$[M+Na]^+$	307.1674	195.1[29]	195.0	-0.1%
**Androstenedione**	C19H24O2	$[M+H]^+$	287.2011	173.4[29]	173.5	0.0%
**Boldenone**	C19H24O2	$[M+H]^+$	287.2011	171.3[29]	170.8	-0.3%
**Boldenone**	C19H24O2	$[M+Na]^+$	309.1831	195.9[29]	195.6	-0.2%
**Epitestosterrone**	C19H28O2	$[M+H]^+$	289.2168	172.7[29]	172.9	0.1%
**Epitestosterrone**	C19H28O2	$[M+Na]^+$	311.1987	197.4[29]	197.4	0.0%
**Testosterone**	C19H28O2	$[M+H]^+$	289.2168	173.6[29]	173.3	-0.2%
**Methyltestosterone**	C20H30O2	$[M+H]^+$	303.2324	177.6[29]	177.8	0.1%
**Mesterolone**	C20H32O2	$[M+H]^+$	305.2481	181.5[29]	182.7	0.7%
**Gestrinone**	C21H24O2	$[M+H]^+$	309.1855	177.0[29]	177.8	0.4%
**Gestrinone**	C21H24O2	$[M+Na]^+$	331.1674	204.1[29]	204.3	0.1%
**Clostebol**	C19H27ClO2	$[M+H]^+$	323.1778	174.8[29]	175.9	0.6%
**Clostebol**	C19H27ClO2	$[M+Na]^+$	345.1597	196.9[29]	196.8	-0.1%
**Stanozolol**	C21H32N2O	$[M+H]^+$	329.2593	191.1[29]	191.4	0.2%
**Oral turinabol**	C20H27ClO2	$[M+Na]^+$	357.1597	200.0[29]	199.5	-0.3%
**Methylclostebol**	C20H29ClO2	$[M+H]^+$	337.1934	179.9[29]	180.2	0.2%
**Methylclostebol**	C20H29ClO2	$[M+Na]^+$	359.1754	201.6[29]	201.4	-0.1%
**Fluoxymestrone**	C20H29FO3	$[M+H]^+$	337.2179	178.6[29]	179.3	0.4%
**Fluoxymestrone**	C20H29FO3	$[M+Na]^+$	359.1998	204.4[29]	203.9	-0.3%
**Danazol**	C22H27NO2	$[M+H]^+$	338.2120	187.1[29]	188.2	0.6%

References

(1) Feuerstein, M. L.; Hann, S.; Causon, T. Chapter 7 Ion Mobility–Time-of-Flight Mass Spectrometry and Applications for Metabolomics. In *Advanced Mass Spectrometry-based Analytical Separation Techniques for Probing the Polar Metabolome*; The Royal Society of Chemistry, 2021; pp 165–184. https://doi.org/10.1039/9781839163524-00165.

(2) Gabelica, V.; Shvartsburg, A. A.; Afonso, C.; Barran, P.; Benesch, J. L. P.; Bleiholder, C.; Bowers, M. T.; Bilbao, A.; Bush, M. F.; Campbell, J. L.; Campuzano, I. D. G.; Causon, T.; Clowers, B. H.; Creaser, C. S.; De Pauw, E.; Far, J.; Fernandez-Lima, F.; Fjeldsted, J. C.; Giles, K.; Groessl, M.; Hogan, C. J., Jr; Hann, S.; Kim, H. I.; Kurulugama, R. T.; May, J. C.; McLean, J. A.; Pagel, K.; Richardson, K.; Ridgeway, M. E.; Rosu, F.; Sobott, F.; Thalassinos, K.; Valentine, S. J.; Wyttenbach, T. Recommendations for Reporting Ion Mobility Mass Spectrometry Measurements. *Mass Spectrom Rev* **2019**, *38* (3), 291–320. https://doi.org/10.1002/mas.21585.

(3) Stow, S. M.; Causon, T. J.; Zheng, X.; Kurulugama, R. T.; Mairinger, T.; May, J. C.; Rennie, E. E.; Baker, E. S.; Smith, R. D.; McLean, J. A.; Hann, S.; Fjeldsted, J. C. An Interlaboratory Evaluation of Drift Tube Ion Mobility-Mass Spectrometry Collision Cross Section Measurements. *Anal Chem* **2017**, *89* (17), 9048–9055. https://doi.org/10.1021/acs.analchem.7b01729.

(4) May, J. C.; McLean, J. A. Ion Mobility-Mass Spectrometry: Time-Dispersive Instrumentation. *Anal Chem* **2015**, *87* (3), 1422–1436. https://doi.org/10.1021/ac504720m.

(5) Paglia, G.; Astarita, G. Metabolomics and Lipidomics Using Traveling-Wave Ion Mobility Mass Spectrometry. *Nature Protocols* **2017**, *12* (4), 797–813. https://doi.org/10.1038/nprot.2017.013.

(6) Campuzano, I. D. G.; Giles, K. Historical, Current and Future Developments of Travelling Wave Ion Mobility Mass Spectrometry: A Personal Perspective. *TrAC Trends in Analytical Chemistry* **2019**, *120*, 115620. https://doi.org/10.1016/j.trac.2019.115620.

(7) Hinnenkamp, V.; Klein, J.; Meckelmann, S. W.; Balsaa, P.; Schmidt, T. C.; Schmitz, O. J. Comparison of CCS Values Determined by Traveling Wave Ion Mobility Mass Spectrometry and Drift Tube Ion Mobility Mass Spectrometry. *Anal. Chem.* **2018**, *90* (20), 12042–12050. https://doi.org/10.1021/acs.analchem.8b02711.

(8) Shvartsburg, A. A.; Smith, R. D. Fundamentals of Traveling Wave Ion Mobility Spectrometry. *Anal. Chem.* **2008**, *80* (24), 9689–9699. https://doi.org/10.1021/ac8016295.

(9) Morsa, D.; Gabelica, V.; De Pauw, E. Fragmentation and Isomerization Due to Field Heating in Traveling Wave Ion Mobility Spectrometry. *J. Am. Soc. Mass Spectrom.* **2014**, *25* (8), 1384–1393. https://doi.org/10.1021/jasms.8b04834.

(10) Hernández-Mesa, M.; D'Atri, V.; Barknowitz, G.; Fanuel, M.; Pezzatti, J.; Dreolin, N.; Ropartz, D.; Monteau, F.; Vigneau, E.; Rudaz, S.; Stead, S.; Rogniaux, H.; Guillarme, D.; Dervilly, G.; Le Bizec, B. Interlaboratory and Interplatform Study of Steroids Collision Cross Section by Traveling Wave Ion Mobility Spectrometry. *Anal. Chem.* **2020**, *92* (7), 5013–5022. https://doi.org/10.1021/acs.analchem.9b05247.

(11) Righetti, L.; Dreolin, N.; Celma, A.; McCullagh, M.; Barknowitz, G.; Sancho, J. V.; Dall'Asta, C. Travelling Wave Ion Mobility-Derived Collision Cross Section for Mycotoxins: Investigating Interlaboratory and Interplatform Reproducibility. *J Agric Food Chem* **2020**, *68* (39), 10937–10943. https://doi.org/10.1021/acs.jafc.0c04498.

(12) Richardson, K.; Langridge, D.; Dixit, S. M.; Ruotolo, B. T. An Improved Calibration Approach for Traveling Wave Ion Mobility Spectrometry: Robust, High-Precision Collision Cross Sections. *Anal. Chem.* **2021**, *93* (7), 3542–3550. https://doi.org/10.1021/acs.analchem.0c04948.

(13) Jeanne Dit Fouque, K.; Fernandez-Lima, F. Recent Advances in Biological Separations Using Trapped Ion Mobility Spectrometry – Mass Spectrometry. *TrAC Trends in Analytical Chemistry* **2019**, *116*, 308–315. https://doi.org/10.1016/j.trac.2019.04.010.

(14) Chai, M.; Young, M. N.; Liu, F. C.; Bleiholder, C. A Transferable, Sample-Independent Calibration Procedure for Trapped Ion Mobility Spectrometry (TIMS). *Anal. Chem.* **2018**, *90* (15), 9040–9047. https://doi.org/10.1021/acs.analchem.8b01326.
(15) Ridgeway, M. E.; Lubeck, M.; Jordens, J.; Mann, M.; Park, M. A. Trapped Ion Mobility Spectrometry: A Short Review. *International Journal of Mass Spectrometry* **2018**, *425*, 22–35. https://doi.org/10.1016/j.ijms.2018.01.006.
(16) Michelmann, K.; Silveira, J. A.; Ridgeway, M. E.; Park, M. A. Fundamentals of Trapped Ion Mobility Spectrometry. *J. Am. Soc. Mass Spectrom.* **2015**, *26* (1), 14–24. https://doi.org/10.1021/jasms.8b04886.
(17) Ridgeway, M. E.; Bleiholder, C.; Mann, M.; Park, M. A. Trends in Trapped Ion Mobility – Mass Spectrometry Instrumentation. *TrAC Trends in Analytical Chemistry* **2019**, *116*, 324–331. https://doi.org/10.1016/j.trac.2019.03.030.
(18) Silveira, J. A.; Michelmann, K.; Ridgeway, M. E.; Park, M. A. Fundamentals of Trapped Ion Mobility Spectrometry Part II: Fluid Dynamics. *J. Am. Soc. Mass Spectrom.* **2016**, *27* (4), 585–595. https://doi.org/10.1021/jasms.8b05241.
(19) Ibrahim, Y. M.; Baker, E. S.; Danielson, W. F., 3rd; Norheim, R. V.; Prior, D. C.; Anderson, G. A.; Belov, M. E.; Smith, R. D. Development of a New Ion Mobility (Quadrupole) Time-of-Flight Mass Spectrometer. *Int J Mass Spectrom* **2015**, *377*, 655–662. https://doi.org/10.1016/j.ijms.2014.07.034.
(20) Hernández-Mesa, M.; Le Bizec, B.; Monteau, F.; García-Campaña, A. M.; Dervilly-Pinel, G. Collision Cross Section (CCS) Database: An Additional Measure to Characterize Steroids. *Anal. Chem.* **2018**, *90* (7), 4616–4625. https://doi.org/10.1021/acs.analchem.7b05117.
(21) R Core Team. *R: A Language and Environment for Statistical Computing*; R Foundation for Statistical Computing: Vienna, Austria, 2021.
(22) RStudio Team. *RStudio: Integrated Development Environment for R*; RStudio, PBC.: Boston, MA, 2020.
(23) Wickham, H.; François, R.; Henry, L.; Müller, K. *Dplyr: A Grammar of Data Manipulation*; 2021.
(24) Wickham, H. *Ggplot2: Elegant Graphics for Data Analysis*; Springer-Verlag New York, 2016.
(25) Schloerke, B.; Cook, D.; Larmarange, J.; Briatte, F.; Marbach, M.; Thoen, E.; Elberg, A.; Crowley, J. *GGally: Extension to “Ggplot2”;* 2021.
(26) Kassambara, A. *Ggpubr: “ggplot2” Based Publication Ready Plots*; 2020.
(27) Xu, S.; Chen, M.; Feng, T.; Zhan, L.; Zhou, L.; Yu, G. Use Ggbreak to Effectively Utilize Plotting Space to Deal With Large Datasets and Outliers. *Frontiers in Genetics* **2021**, *12*, 2122. https://doi.org/10.3389/fgene.2021.774846.
(28) Davis, D. E.; Leaptrot, K. L.; Koomen, D. C.; May, J. C.; Cavalcanti, G. de A.; Padilha, M. C.; Pereira, H. M. G.; McLean, J. A. Multidimensional Separations of Intact Phase II Steroid Metabolites Utilizing LC–Ion Mobility–HRMS. *Anal. Chem.* **2021**, *93* (31), 10990–10998. https://doi.org/10.1021/acs.analchem.1c02163.
(29) Velosa, D. C.; Rivera, M. E.; Neal, S. P.; Olsen, S. S. H.; Burkus-Matesevac, A.; Chouinard, C. D. Toward Routine Analysis of Anabolic Androgenic Steroids in Urine Using Ion Mobility-Mass Spectrometry. *J. Am. Soc. Mass Spectrom.* **2022**, *33* (1), 54–61. https://doi.org/10.1021/jasms.1c00231.
(30) Causon, T. J.; Si-Hung, L.; Newton, K.; Kurulugama, R. T.; Fjeldsted, J.; Hann, S. Fundamental Study of Ion Trapping and Multiplexing Using Drift Tube-Ion Mobility Time-of-Flight Mass Spectrometry for Non-Targeted Metabolomics. *Analytical and Bioanalytical Chemistry* **2019**, *411* (24), 6265–6274. https://doi.org/10.1007/s00216-019-02021-8.
(31) Hernández-Mesa, M.; Monteau, F.; Le Bizec, B.; Dervilly-Pinel, G. Potential of Ion Mobility-Mass Spectrometry for Both Targeted and Non-Targeted Analysis of Phase II Steroid Metabolites in Urine. *Analytica Chimica Acta: X* **2019**, *1*, 100006. https://doi.org/10.1016/j.acax.2019.100006.
